# Major flowering time genes of barley: allelic diversity, effects, and comparison with wheat

**DOI:** 10.1007/s00122-021-03824-z

**Published:** 2021-05-09

**Authors:** Miriam Fernández-Calleja, Ana M. Casas, Ernesto Igartua

**Affiliations:** Department of Genetics and Plant Production, Aula Dei Experimental Station, EEAD-CSIC, Avenida Montañana, 1005, 50059 Zaragoza, Spain

## Abstract

**Key message:**

This review summarizes the allelic series, effects, interactions between genes and with the environment, for the major flowering time genes that drive phenological adaptation of barley.

**Abstract:**

The optimization of phenology is a major goal of plant breeding addressing the production of high-yielding varieties adapted to changing climatic conditions. Flowering time in cereals is regulated by genetic networks that respond predominately to day length and temperature. Allelic diversity at these genes is at the basis of barley wide adaptation. Detailed knowledge of their effects, and genetic and environmental interactions will facilitate plant breeders manipulating flowering time in cereal germplasm enhancement, by exploiting appropriate gene combinations. This review describes a catalogue of alleles found in QTL studies by barley geneticists, corresponding to the genetic diversity at major flowering time genes, the main drivers of barley phenological adaptation: *VRN-H1* (*HvBM5A*), *VRN-H2* (*HvZCCTa-c*), *VRN-H3* (*HvFT1*), *PPD-H1* (*HvPRR37*), *PPD-H2* (*HvFT3*), and *eam6/eps2* (*HvCEN*). For each gene, allelic series, size and direction of QTL effects, interactions between genes and with the environment are presented. Pleiotropic effects on agronomically important traits such as grain yield are also discussed. The review includes brief comments on additional genes with large effects on phenology that became relevant in modern barley breeding. The parallelisms between flowering time allelic variation between the two most cultivated *Triticeae* species (barley and wheat) are also outlined. This work is mostly based on previously published data, although we added some new data and hypothesis supported by a number of studies. This review shows the wide variety of allelic effects that provide enormous plasticity in barley flowering behavior, which opens new avenues to breeders for fine-tuning phenology of the barley crop.

**Supplementary Information:**

The online version contains supplementary material available at 10.1007/s00122-021-03824-z.

## Introduction

Phenological adjustment is critical for maximizing yields during crop adaptation. Synchronizing the plant cycle to the prevailing environmental conditions was key to enable the expansion of crops to agricultural environments far distant from those found in their progenitors’ domestication centers (Evans 1996; Knüpffer et al. 2003; Cockram et al. 2007b; Zohary et al. 2012). Currently, plant breeders are challenged to develop new cultivars allowing a profitable production under increasingly unfavorable and shifting environmental conditions, due to climate change (Verstegen et al. 2014). Under these circumstances, the timing of the developmental milestones, with flowering first and foremost, is essential to achieve adaptation to increasingly prevalent temperature and water deficit stresses (Rosenzweig et al. 2001; Kazan and Lyons 2016). Fine-tuning crop phenology will be critical to reduce the impacts of these limiting factors on yield, minimizing the exposure of the most sensitive growth stages to climate extremes (Craufurd and Wheeler 2009).

Barley (*Hordeum vulgare* L.) represents a relevant model for agroecological adaptation since it has been cultivated in all temperate regions from the Arctic Circle to the tropics (Ullrich 2011). Besides, it belongs to the *Triticeae* tribe, an economically and socially important group of species providing a significant share of food and feed (Al-Saghir 2016).

**Fig. 1:**
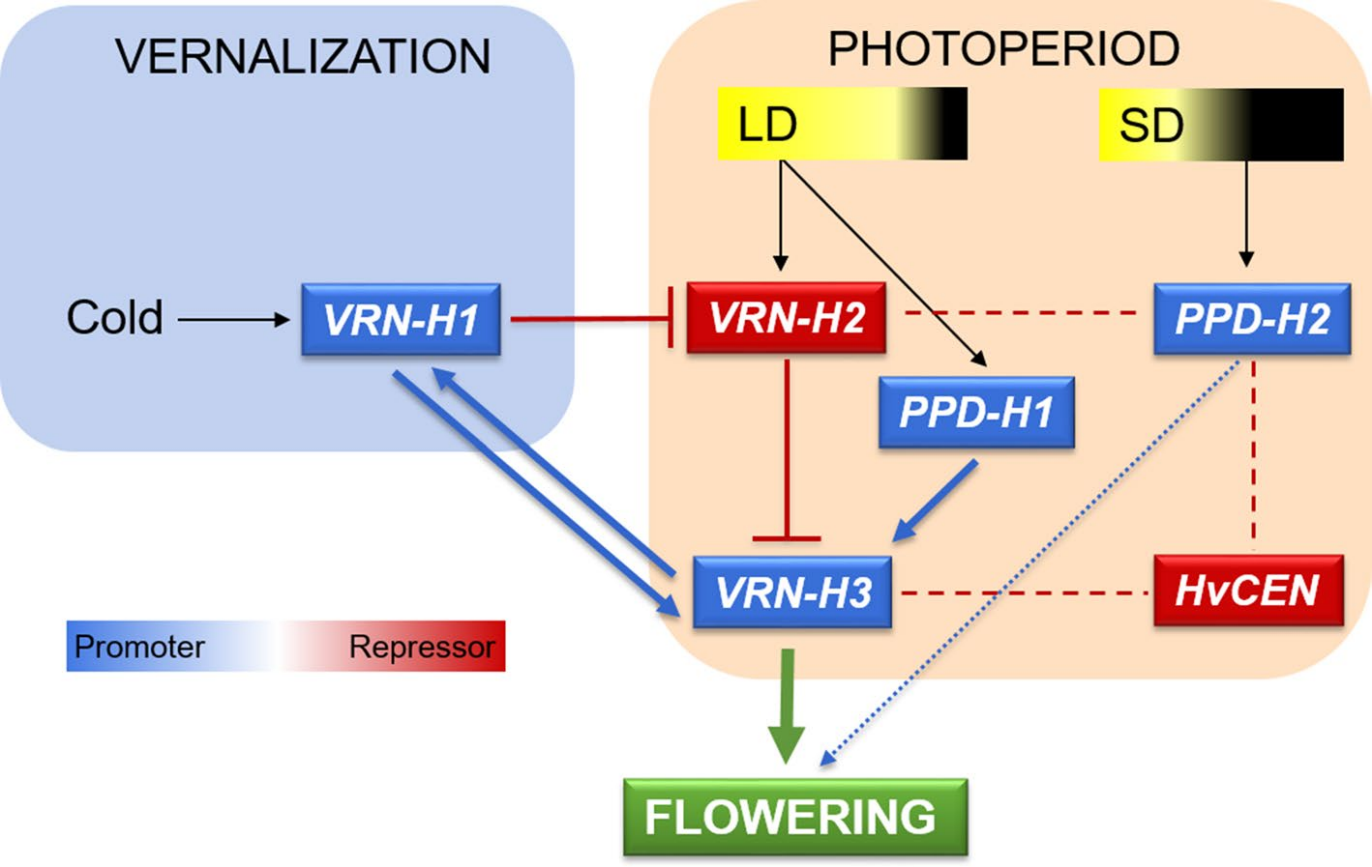
Flowering time control in barley: main genes, environmental cues and regulatory pathways. Reproductive transition in barley is regulated by genetic networks that respond to extended periods of low temperature (vernalization, blue frame) and day length (photoperiod, orange frame). Genes depicted in blue promote flowering, whereas genes depicted in red act as repressors. Blue and green arrows indicate induction. Red lines with blunt ends indicate repression. Antagonistic relationships between genes reported in the literature are represented as dashed red lines. *PPD-H2* connection with flowering is represented as a dashed blue line because it induces spikelet initiation but not floral development (Mulki et al. 2018). *LD* long days, *SD* short days

Flowering time is a complex trait, tightly controlled by genetic networks that integrate environmental cues. In barley, the transition to the reproductive stage is mainly controlled by genes affected by two main seasonal cues (Laurie 2009): day length (photoperiod) and extended periods of low temperature (vernalization) (Fig. 1). The allelic richness at these genes is the basis for barley wide adaptation (Campoli and von Korff 2014). A thorough understanding of the genetic and environmental control of flowering time, and better knowledge and utilization of the genetic diversity, will enable breeders to develop cultivars adapted to specific areas and climates, by deploying appropriate phenology gene combinations (Wilczek et al. 2010; Nazim Ud Dowla et al. 2018).

Depending on the vernalization requirement, barley cultivars are roughly classified as having winter or spring growth habit, although this scale is oversimplified, as we will see later on. “Winter” varieties are usually sown in autumn and need vernalization for timely flowering. This adaptive feature delays apex transition, preventing the exposure of frost-sensitive floral organs to freezing winter temperatures, ensuring flowering occurs only under warm conditions, in spring. Spring types are sown in spring, in regions with too harsh winters, and show null or reduced vernalization requirement. Almost all wild barleys are winter type, so one of the prerequisites for barley production expansion to spring sowing areas was the development of lines lacking vernalization requirements (Pourkheirandish and Komatsuda 2007). The geographical distribution of winter and spring varieties is mainly mediated by winter harshness, although the need to avoid unfavorable conditions for grain filling at the end of the season is also determinant in the Mediterranean region (Yahiaoui et al. 2008; Verstegen et al. 2014). In addition to temperature, flowering time also depends on photoperiod (Laurie 1997). In wild barleys, photoperiods over 12 h trigger a rapid switch to reproductive growth, a phenomenon called photoperiod sensitivity. This behavior was also typical of the first domesticated barleys, and slowed down their spread to areas with winter temperatures too low for barley to survive. In these areas, spring sowing was the only option, and photoperiod sensitivity reduced vegetative growth to a minimum over spring and summer, insufficient to attain acceptable agronomic performance. Therefore, photoperiod insensitivity enabled the expansion of barley cultivation into higher latitudes (Komatsuda 2014).

The purpose of this review is to describe the catalogue of alleles found in QTL studies by barley geneticists, which likely correspond to the genetic diversity at major flowering time genes. We will summarize the diversity found associated with *VRN*-*H1* (*HvBM5A*), *VRN*-*H2* (*HvZCCTa*-*c*), *VRN*-*H3* (*HvFT1*), *PPD*-*H1* (*HvPRR37*), *PPD*-*H2* (*HvFT3*), and *eam6*/*eps2* (*HvCEN*), as the main drivers of phenological adaptation of barley during its long history of expansion starting in the Neolithic. We will also cover briefly some genes that have become relevant in modern barley breeding, with large effects on phenology, namely, *denso*, *eam8* (*EARLY FLOWERING3* or *HvELF3*) and *eam5* (*HvPHYTOCHROME C* or *HvPHYC*). In addition, we will outline parallelisms, differences of the main flowering time genes, and allelic variation between the most important *Triticeae* cultivated species, barley and wheat (*Triticum* species).

Two disclaimers are needed. First, heading date has been commonly used as a surrogate for flowering time in barley, although this equivalence is not fully correct (Alqudah and Schnurbusch 2017). Different authors have used slightly different methods to record the moment of “flowering”. The most common has been the recording of awn tipping (Z49) and heading (Z55), according to the Zadoks growth scale (Zadoks et al. 1974). For the sake of simplicity, and to facilitate communication, “heading” and “flowering”, are used as synonyms in this article. The slight differences of timing of occurrence between those physiological stages do not affect the purpose of this review. Second, in QTL studies, it is almost impossible to be certain about the actual gene underlying each effect detected. However, authors make informed guesses which, in most major flowering time genes cases, are eventually confirmed with functional proofs. We have summarized QTL studies following the authors’ judgment regarding underlying genes. When QTL detection preceded the declaration of candidate genes in the region, we have used the later literature or our own judgment to declare possible underlying major genes.

## Vernalization response

The genetic control of vernalization in winter barley is based on three genes: *VRN-H1* (Yan et al. 2003; Trevaskis et al. 2003), *VRN-H2* (Yan et al. 2004b), and *VRN-H3* (Yan et al. 2006), which take part in a feedback regulatory loop through epistatic interactions (Distelfeld et al. 2009a) (Fig. 1). According to the currently accepted model, the high levels of *VRN-H2* during the long days of fall repress flowering by preventing the expression of *VRN-H3*, which limits the up-regulation of *VRN-H1*. The up-regulation of *VRN-H1* during winter results in the down-regulation of *VRN-H2*, the release of *VRN-H3* from its repression and, under long days, the *VRN-H3* up-regulation of *VRN-H1* transcripts beyond the threshold required to initiate flowering. Loss of *VRN-H2* results in earlier expression of *VRN-H3* under long-day conditions, and promotion of flowering without vernalization (Trevaskis et al. 2006; Distelfeld et al. 2009a).

### *VRN-H1*

*VRN-H1* is the central regulator of vernalization-induced flowering in barley (Trevaskis et al. 2007; Distelfeld et al. 2009a). In winter cultivars (with an active *VRN-H2* allele), the expression of this gene is induced by vernalization and accelerates flowering by the promotion of inflorescence initiation at the shoot apex (Trevaskis et al. 2003). *VRN-H1* encodes an *AP1*-*like* MADS-box transcription factor and is located on chromosome 5HL. In winter cultivars, a prolonged cold period induces *VRN-H1* transcription, eventually leading to phase transition from vegetative to reproductive growth (Yan et al. 2003; Danyluk et al. 2003; Trevaskis et al. 2003). Activation of *VRN-H1* is quantitative, with longer cold treatments inducing higher levels of expression (Yan et al. 2003; Danyluk et al. 2003; Trevaskis et al. 2003; von Zitzewitz et al. 2005; Sasani et al. 2009), which results in earlier transition to the reproductive phase (Sasani et al. 2009). The vernalization-induced transcription of *VRN-H1* is mediated by epigenetic regulation involving changes in chromatin state, through particular modifications in the pattern of histone methylation, whose maintenance provides a memory of cold exposure in winter barley plants (Oliver et al. 2009). Deng et al. (2015) identified binding targets of the VRN1 protein and demonstrated that it regulates flowering repressors *OS2* and *VRN-2*, and flowering promoter *VRN-3*. VRN1 also binds to the promoters of *CBF* (*C-repeat Binding Factor*) genes that play critical roles in low-temperature induction of freezing tolerance and to *VRS1*, which regulates spike architecture. Thus, in addition to controlling flowering, VRN1 directly targets genes in pathways that control other key traits such as frost tolerance.

The previous paragraph describes the classic hypothesis, which still holds, but there is evidence of the presence of a wide allelic diversity at this gene, with more nuanced phenotypic effects. The wild-type *vrn-H1* allele, found in winter barleys, is induced by cold exposure and development, and is characterized by an intact first intron. Other reported alleles differ in the first intron structure, containing deletions or insertions, which affect the length of the cold period needed to reach full de-methylation of the gene (Fu et al. 2005; von Zitzewitz et al. 2005; Cockram et al. 2007a; Hemming et al. 2009). While this is the main regulatory mechanism of this gene, there may be more. Recently, the presence of additional intron regulatory elements in *VRN-H1,* differentiating winter, spring, and wild barleys, has been advocated (Wiegmann et al. 2019). Hemming et al. (2009) characterized at least eleven different alleles based on the size of the first intron (11 kb in the wild-type *vrn-H1*) (Table S1). Alleles characterized by insertions or large deletions within *VRN-H1* intron 1, that disrupt putative cis-regulatory regions presumably required for repression of *VRN-H1*, are associated with increased *VRN-H1* transcript levels, and with earlier flowering without vernalization. In contrast, alleles lacking small segments of the intron, have been associated with moderate basal transcript levels and a weaker flowering stimulation (Szűcs et al. 2007; Hemming et al. 2009; Casao et al. 2011a; Oliver et al. 2013). Therefore, the various *VRN-H1* alleles display a continuum gradation in the strength of flowering promotion (Takahashi and Yasuda 1971; Szűcs et al. 2007). Regarding the gene action of the *VRN-H1* allelic series, the accepted model states that the winter allele is recessive, while the rest are dominant (Takahashi and Yasuda 1971; Haas et al. 2020), although additive effects in F_1_ crosses have been observed for non-strict spring alleles (M. Fernández-Calleja, unpublished).

The vernalization requirement determines the cultivar adaptation range in barley. Mutations in *VRN-H1* and the loss of strong cold requirements allowed the expansion of cultivated barley to areas where spring types are more suitable (von Bothmer et al. 2003; Cockram et al. 2011), although this explanation can be extended to encompass the role of less strict winter types, adapted to fall sowings in areas with warm winters. In fact, several studies have reported ample allelic variation at *VRN-H1* and its relation with geographical distribution, in accordance with this hypothesis (Cockram et al. 2007a, b; Saisho et al. 2011; Zhang et al. 2015a, b; Dondup et al. 2016; Contreras‐Moreira et al. 2019). Besides vernalization response, the *VRN-H1* region has also been associated with winter survival in the field and frost tolerance (Francia et al. 2004; Cuesta-Marcos et al. 2015), with deep implications on the geographical distribution of barley cultivars. In autumn-sown trials subjected to frost stress, the winter *vrn-H1* frost-resistance allele provided a yield advantage (Tondelli et al. 2014). Recently, Rizza et al. (2016) established that the structure of *VRN-H1* intron 1 was strongly correlated not only with vernalization response but also with frost tolerance. In general terms, the higher the vernalization requirement, the higher the frost tolerance levels. However, this is not always true. Some alleles inducing similar vernalization response were associated with different levels of frost tolerance. The alleles *VRN-H1-1*, *VRN-H1-2*, *VRN-H1-3*, and *VRN-H1-4* all showed similarly low frost tolerance levels. The alleles *VRN-H1-6* (medium–high vernalization requirement, Casao et al. 2011b), and *vrn-H1 (5200)* (high vernalization requirement) showed medium–high levels of frost tolerance, whereas allele *vrn-H1 (5300)* was associated with a higher level of frost tolerance. In principle, *vrn-H1 (5200)* and *vrn-H1 (5300)*, which are differentiated by partial amplifications of the first intron, are considered functionally similar variants of the wild-type winter allele, both displaying a high vernalization requirement. However, they present sequence differences; *vrn-H1 (5200)* has a small deletion (118 bp) of a region including a MITE (miniature inverted-repeat transposable element), which could affect epigenetic regulation (von Zitzewitz et al. 2005). In fact, these apparent discrepancies between vernalization and frost tolerance may be a result of lack of experiments run at the sensitivity needed to discriminate all the effects on both traits. Interestingly, from a breeding point of view, Casao et al. (2011a) demonstrated that it is possible to manipulate vernalization requirement with only minor effects on frost tolerance, by taking advantage of the known interaction between *VRN-H1*/*Fr-H1* and *Fr-H2* (Galiba et al. 2009; Dhillon et al. 2010). This finding opens the path to breed new cultivars that are better suited to a range of winter harshness, especially in a climate change scenario, by combining reduced vernalization requirement alleles and the frost-resistant *Fr-H2* allele from strict winter lines.

An interesting hypothesis argues that vernalization, despite its well-proven adaptive role, could carry an agronomic burden when sowing dates are uncertain. Under these circumstances, frost-tolerant facultative cultivars could be advantageous (Muñoz-Amatriaín et al. 2020).

To summarize the results of flowering time QTL in the *VRN-H1* region (Table 1), we followed the terminology of Hemming et al. (2009) for the allelic series (Table S1). This region has been strongly associated with vernalization response in controlled conditions experiments in which, in the absence of cold, the winter *vrn-H1* allele consistently delayed flowering (Laurie et al. 1995; Cuesta-Marcos et al. 2008b; Karsai et al. 2008). There is evidence of gradually decreasing vernalization responses of alleles *VRN-H1-6* (Casao et al. 2011a) and *VRN-H1-4* (Casao et al. 2011a, b). The late-flowering effect of the winter *vrn-H1* allele was also found in field trials, apparently when the conditions prevent the completion of the vernalization requirement (e.g., spring sowings) (Laurie et al. 1995; Francia et al. 2004; Cuesta-Marcos et al. 2008b; Tondelli et al. 2014), although the actual measurement of the vernalization potential in field trials is rare. Some studies were sensitive enough to reveal phenotypic differences between *VRN-H1* alleles with more similar vernalization requirements (Cuesta-Marcos et al. 2008a; Rollins et al. 2013; Afsharyan et al. 2020). Genome wide association studies (GWAS) carried out on large germplasm collections also detected important associations between *VRN-H1* and flowering time, of the same kind as for biparental populations (Table 1).

**Table 1:** *VRN-H1* polymorphisms and effects on flowering

Population	Environment/conditions^a^	*VRN-H1* allele^b^		*VRN-H2* segregating^c^	Additive effect^d^
		Parent 1	Parent 2		
*Biparental populations*					
Igri × Triumph^1^	Controlled conditions	*vrn-H1*	**VRN-H1-3**	Yes	
Igri × Triumph^1^	Field, spring sowing	*vrn-H1*	**VRN-H1-3**	Yes	1.10 days
Dicktoo × Morex^2^	Controlled conditions, uv	*vrn-H1*	**VRN-H1-1**	No	9.00–24.00 days
Mogador × Beka^3^	Controlled conditions	*vrn-H1*	**VRN-H1-1**	Yes	0.20–1.20 leaves
Mogador × Beka^3^	Field, spring sowing	*vrn-H1*	**VRN-H1-1**	Yes	7.30–10.20 days
Mogador × Beka^3^	Field, winter sowing	*vrn-H1*	**VRN-H1-1**	Yes	0.80 days
Nure × Tremois^4^	Field, spring sowing	*vrn-H1*	**VRN-H1-7**	Yes	2.30 days
Nure × Tremois^4^	Field, winter sowing	*vrn-H1*	**VRN-H1-7**	Yes	0.90 days
Arta × Keel^5^	Field, winter sowing	*VRN-H1-6*	**VRN-H1-4**	Yes	1.10–6.50 days
Arta × Keel^5^	Field, autumn sowing	*VRN-H1-6*	**VRN-H1-4**	Yes	0.30–1.00 days
Plaisant × Orria^6^	Field, winter sowing	*vrn-H1*	**VRN-H1-4**	No	3.70 days
Plaisant × Orria^6^	Field, autumn sowing	*vrn-H1*	**VRN-H1-4**	No	0.80–1.20 days
Plaisant × (Candela × 915006)^7^	Controlled conditions, uv	*vrn-H1*	**VRN-H1-4**	Yes	11.60 days
*GWAS*					
HEB-25^8^	Field, spring sowing	Wild	**VRN-H1-3**	Yes	3.80 days
HEB-25^9^	Field, winter sowing	Wild	**VRN-H1-3**	Yes	3.00 days
HEB-25^10^	Field, autumn sowing	Wild	**VRN-H1-3**	Yes	2.70 days
HEB-YIELD^11^	Field, spring sowing	Wild	**VRN-H1-3**	Yes	ns
HEB-YIELD^11^	Field, winter sowing	Wild	**VRN-H1-3**	Yes	2.50 days
HEB-YIELD^11^	Field, autumn sowing	Wild	**VRN-H1-3**	Yes	2.20 days
Phenology diversity panel^12, 13^	Field, autumn sowing			Yes	6.30 days
MAGIC^14^	Field, spring sowing	*VRN-H1-6*	**VRN-H1-3**	No	2.70 days

Surveys in which associations between flowering time and the *VRN-H1* locus region were detected are reported. It includes linkage mapping studies performed in biparental populations segregating for *VRN-H1*, and genome wide association analyses

^a^Environmental conditions (*uv* unvernalized), ^b^*VRN-H1* alleles, ^c^*VRN-H2* segregation state in the population, and ^d^*VRN-H1* additive effect were collected from the original sources (*ns* nonsignificant effect). ^b^Alleles contributing to earliness are highlighted in bold

^1^Laurie et al. (1995), ^2^Karsai et al. (2008), ^3^Cuesta-Marcos et al. (2008b), ^4^Tondelli et al. (2014), ^5^Rollins et al. (2013), ^6^Mansour et al. (2014), ^7^Malosetti et al. (2011), ^8^Maurer et al. (2015), ^9^Saade et al. (2016), ^10^Merchuk-Ovnat et al. (2018), ^11^Wiegmann et al. (2019), ^12^He et al. (2019), ^13^Hill et al. (2019), ^14^Afsharyan et al. (2020)

The adaptive role of *VRN-H1* is confirmed by its influence on yield and yield-related traits (Wang et al. 2010; Rollins et al. 2013; Mansour et al. 2014; Tondelli et al. 2014). The study of Rollins et al. (2013) showed that in short-season environments, faster development associated with low vernalization requirement alleles was beneficial for yield. These results are in agreement with those from Mansour et al. (2014) and Tondelli et al. (2014), who found an important QTL by environment interaction at *VRN-H1* for grain yield. In the population Nure (*vrn-H1*) x Tremois (*VRN-H1-7*), a positive contribution on grain yield was reported for the winter allele of Nure in autumn-sown trials, whereas opposite results were found in the late sowing sites (Tondelli et al. 2014). In the case of the population Orria (*VRN-H1-4*) x Plaisant (*vrn-H1*), the winter *vrn-H1* allele from Plaisant reduced grain yield significantly at the three trials which experienced higher temperatures (Mansour et al. 2014). On the contrary, no effect of *VRN-H1* on grain yield was found in a study carried out under similar Mediterranean conditions with the spring x winter population Beka x Mogador (Cuesta-Marcos et al. 2009). In this last case, all trials were sown in autumn and vernalization requirements were probably fulfilled. From the latter studies, it seems clear that the winter *vrn-H1* allele is detrimental for yield at warm sites prone to terminal stress (probably by not meeting the vernalization requirements on time).

Most recently, Voss-Fels et al. (2018) reported that natural allelic variation at *VRN-H1* modulates root growth angle and root length. Compared to the wild-type allele, spring alleles in barley were associated with reduced root elongation and maximum root length between anthesis and maturity. Therefore, the authors suggested a role for this gene in the adaptation of barley to drought. Multi-parental population studies are also a relevant source of evidence for the pleiotropic effects of *VRN-H1* on multiple agronomic traits (Maurer et al. 2016; Saade et al. 2016; Nice et al. 2017; Sharma et al. 2018; Wiegmann et al. 2019). Abdel-Ghani et al. (2019) identified the *VRN-H1* region as hotspot controlling shoot and root architecture under osmotic stress in a spring barley collection. These findings are in agreement with Rollins et al. (2013) and Voss-Fels et al. (2018), who reported *VRN-H1* as an important region under drought conditions, with pleiotropic effects on root architecture, biomass and yield. When the nested association mapping (NAM) population HEB-25 (Halle Exotic Barley) was evaluated with salt stress in field conditions, wild alleles at the *VRN-H1* locus increased height, reduced harvest index, grains per ear and yield under stress and control treatments (Saade et al. 2016). The yield reduction effect of the wild *vrn-H1* alleles was associated with a decreased number of ears but larger grains, supported by Sharma et al. (2018) findings.

The *VRN-H1* region is involved in epistatic interactions affecting heading time and other agronomic traits. A combination of the winter *vrn-H1* allele and the insensitive *ppd-H1* allele resulted in the latest flowering genotypes in a population segregating for both genes (Karsai et al. 2008). Besides, the most significant epistatic interaction under a high temperature conditions experiment (foil tunnel) was among regions that corresponded to *VRN-H3* and *VRN-H1* (Afsharyan et al. 2020). Several studies have found a significant interaction between *VRN-H1* and *HvCEN*, with effects on heading time and yield (Laurie et al. 1995; Cuesta-Marcos et al. 2008b; Mansour et al. 2014; Boudiar et al. 2016), reviewed below in the ‘*HvCEN*’ section. Although probably the most important interaction in which *VRN-H1* is involved is that with the repressor *VRN-H2*, reviewed in the next section, devoted to that gene.

In wheat, *VRN-1* presents homoeologous copies in chromosomes 5A, 5B and 5D. Polymorphisms at this gene are richer in wheat than in barley. Besides deletions in the first intron (Fu et al. 2005), like in barley, many mutations have been described in other regulatory regions and coding sequence, all associated with increased expression of the gene and accelerated flowering in the absence of vernalization (Yan et al. 2003, 2004a; Chu et al. 2011; Li et al. 2013; Muterko et al. 2015; Zhang et al. 2015a, b; Kippes et al. 2018). These mutations give rise to spring dominant alleles, with the *VRN-A1* allele showing the strongest effect on flowering time (lack of vernalization requirement), and *VRN-B1* and *VRN-D1* alleles showing a weaker effect (reduced vernalization requirement) (Trevaskis et al. 2003). Moreover, copy number variation has also been described for *VRN-1* in subgenome A, influencing vernalization requirement duration and flowering time of wheat (Díaz et al. 2012; Li et al. 2013; Würschum et al. 2015; Dixon et al. 2019). Besides, the translocation of the region from chromosome 5A that contains the *VRN-1* gene to the chromosome 5DS gave rise to the gene *VRN-D4*, which also reduces vernalization requirement (Kippes et al. 2015).

Summarizing, *VRN-H1* is the major flowering promoter in the vernalization pathway. It is induced by cold exposure and development. There is a large number of *VRN-H1* alleles, which are defined by the length of the first intron, and present a whole gradation of responses to vernalization, from strict winter to spring growth habits. *VRN-H1* effect on flowering time is mainly detected when vernalization requirements are not fully satisfied or are met too late. *VRN-H1* has a wide influence on barley agronomics, through extensive pleiotropic effects (frost tolerance, root architecture, yield…), revealing an adaptive role beyond flowering. The direction and magnitude of *VRN-H1* effects on grain yield vary depending on the environment, particularly on a delicate balance between *VRN-H1* allele, probability of frost occurrence, and vernalizing potential.

### *VRN-H2*

*VRN-H2* is the central flowering repressor of the vernalization mechanism. When active, it delays flowering until plants have satisfied their cold needs, when *VRN-H1* represses it (Laurie et al. 1995; Yan et al. 2004b). This epistatic system is clearly a major factor controlling the time to flowering in winter barley (Yan et al. 2003; von Zitzewitz et al. 2005). It has been validated in genetic studies with biparental populations (Karsai et al. 2005; Kóti et al. 2006; Szűcs et al. 2007) and is supported by the results observed in a number of QTL studies (Cuesta-Marcos et al. 2008a, b; Malosetti et al. 2011; Maurer et al. 2015). Recently, ChIP-seq analyses have confirmed the direct regulation of *VRN-H2* by *VRN-H1* (Deng et al. 2015).

*VRN-H2* encodes a cluster of three *ZCCT*-*H* genes, which contain a zinc finger and a *CONSTANS*-like domain, and are located on chromosome 4HL. Functional diversity at *VRN-H2* is the result of the presence or absence of the whole *ZCCT-H* gene cluster (Karsai et al. 2005) (Table S1). Winter barleys carry the functional dominant allele (Distelfeld et al. 2009a). The null recessive allele of *VRN-H2* largely bypasses the requirement for vernalization and causes early flowering, regardless of the allelic state at *VRN-H1*. The facultative growth habit is the result of the deletion of the *VRN-H2* locus and the presence of a winter *vrn-H1* allele. These cultivars show winter hardiness but lack an obligate vernalization requirement (Dubcovsky et al. 2005; Karsai et al. 2005; von Zitzewitz et al. 2005). Recent results suggest that facultative barleys, with very high frost tolerance, may contain full or partial deletions of some of the *HvZCCT* genes (Muñoz-Amatriaín et al. 2020).

Its high expression is only achieved in long days (Yan et al. 2004b; Karsai et al. 2005; Trevaskis et al. 2006). However, it has been recently reported that it is also expressed, at lower levels, at day lengths below 12 h (Monteagudo et al. 2019b), or under conditions in which plants are deceived to sense that they are in long days (Turner et al. 2013). Therefore, this gene is not under the direct control of the light-sensing mechanism, but is instead under the control of clock-regulated downstream components (Turner et al. 2013; Mulki and von Korff 2016).

Actually, the regulation of *VRN-H2* is not fully unravelled. Besides its repression by *VRN-H1*, recent shreds of evidence indicate that high expression of *VRN-H2* necessitates of long days and induction by *HvCO1*/*CO2*, the barley orthologues of the *Arabidopsis CONSTANS* (*CO*) gene, and *PPD-H1.* The VRN2 protein is instrumental in the repression of *VRN-H3* and, hence, of flowering in winter barley, before vernalization (Mulki and von Korff 2016). In addition, Casao et al. (2011b) suggested that *VRN-H2* could also down-regulate *PPD-H2* expression under long days. The antagonism between the expression of these two genes is clear, but the direction of the repression is not.

There is ample evidence on the presence of flowering time QTL in the region of *VRN-H2*, in a variety of barley biparental populations and association panels (Table 2). In general, no effect was detected in fully vernalized experiments, whereas QTL were detected when vernalization was not complete, under long days, and not under short days. This agrees with the dynamics of its expression explained above. *VRN-H2* presents a broad range of additive effects on flowering time, detected in spring-sown trials. It depends on the presence of at least a winter *VRN-H1* allele in the population, which causes wide segregation of vernalization requirements, and on the sowing date and location, which determines the degree of vernalization fulfilment. Karsai et al. (2006) found that the effect of *VRN-H2* on flowering time became significant when the photoperiod was 12 h or longer, which agrees with the day-length threshold leading to a marked rise in *VRN-H2* expression that Monteagudo et al. (2019b) determined, and was suggested as the deadline to fulfil the vernalization requirement in winter barley. However, some studies have detected flowering QTL on the *VRN-H2* region under conditions apparently non-inductive for this gene, like vernalized plants (Karsai et al. 2005, 2006, 2008), possibly due to an incomplete vernalization treatment (6 weeks) (Table 2). Also, a subtle but consistent effect in short days has been reported (Laurie et al. 1995; Karsai et al. 2005, 2006; Cuesta-Marcos et al. 2008b; Rollins et al. 2013) (Table 2).

**Table 2:** *VRN-H2* polymorphisms and effects on flowering

Population	Environment/conditions^a^	Vernalization^b^	Photoperiod^c^	*VRN-H2* allele^d^		*VRN-H1* segregating^e^	Additive effect^f^
				Parent 1	Parent 2		
*Biparental populations*							
Igri × Triumph^1^	Controlled conditions	6–0w	16 h	*VRN-H2*	**vrn**-**H2**	Yes	
Igri × Triumph^1^	Field, spring sowing		LD	*VRN-H2*	**vrn**-**H2**	Yes	1.00 days
Igri × Triumph^1^	Field, autumn sowing		SD	*VRN-H2*	**vrn**-**H2**	Yes	0.90 days
Kompolti Korai × Dicktoo^2^	Controlled conditions	Null	8 h	*VRN-H2*	**vrn**-**H2**	No	4.50 days
Kompolti Korai × Dicktoo^2^	Controlled conditions	Null	16 h	*VRN-H2*	**vrn**-**H2**	No	12.20 days
Kompolti Korai × Dicktoo^2^	Controlled conditions	Incomplete (6w)	16 h	*VRN-H2*	**vrn**-**H2**	No	3.30 days
Kompolti Korai × Dicktoo^2^	Field, spring sowing		LD	*VRN-H2*	**vrn**-**H2**	No	1.70 days
Kompolti Korai × Dicktoo^3^	Controlled conditions	Incomplete (6w)	10 h	*VRN-H2*	**vrn**-**H2**	No	3.00 days
Kompolti Korai × Dicktoo^3^	Controlled conditions	Incomplete (6w)	12 h	*VRN-H2*	**vrn**-**H2**	No	13.50 days
Kompolti Korai × Dicktoo^3^	Controlled conditions	Incomplete (6w)	14 h	*VRN-H2*	**vrn**-**H2**	No	12.40 days
Kompolti Korai × Dicktoo^3^	Controlled conditions	Incomplete (6w)	16 h	*VRN-H2*	**vrn**-**H2**	No	15.80 days
Kompolti Korai × Dicktoo^3^	Controlled conditions	Incomplete (6w)	18 h	*VRN-H2*	**vrn**-**H2**	No	17.40 days
Kompolti Korai × Dicktoo^4^	Controlled conditions	Incomplete (6w)	24 h, constant Tª	*VRN-H2*	**vrn**-**H2**	No	12.00 days
Kompolti Korai × Dicktoo^4^	Controlled conditions	Incomplete (6w)	16 h, constant Tª	*VRN-H2*	**vrn**-**H2**	No	12.00 days
Kompolti Korai × Dicktoo^4^	Controlled conditions	Incomplete (6w)	16 h, termocycle	*VRN-H2*	**vrn**-**H2**	No	7.00 days
Mogador × Beka^5^	Controlled conditions	Complete (8w)	10 h	*VRN-H2*	**vrn**-**H2**	Yes	0.40 leaves
Mogador × Beka^5^	Controlled conditions	Null	17 h	*VRN-H2*	**vrn**-**H2**	Yes	1.10 leaves
Mogador × Beka^5^	Field, spring sowing		LD	*VRN-H2*	**vrn**-**H2**	Yes	3.6–6.3 days
Mogador × Beka^5^	Field, winter sowing		SD	*VRN-H2*	**vrn**-**H2**	Yes	0.50 days
17 interconnected populations^6^	Controlled conditions	Null	17 h	*VRN-H2*	**vrn**-**H2**	Yes	2.00 leaves
17 interconnected populations^6^	Controlled conditions	Complete (8w)	17 h	*VRN-H2*	**vrn**-**H2**	Yes	0.70 leaves
17 interconnected populations^6^	Field, winter sowing		LD	*VRN-H2*	**vrn**-**H2**	Yes	0.70 days
ISR42-8 × Scarlett^7^	Field, spring sowing		LD	*VRN-H2*	**vrn**-**H2**	Yes	0.70 days
Nure × Tremois^8^	Field, spring sowing		LD	*VRN-H2*	**vrn**-**H2**	Yes	1.20 days
KNG × Azumamugi^9^	Field, spring sowing		LD	*VRN-H2*	**vrn**-**H2**	Yes	7.10 days
Arta × Keel^10^	Field, autumn sowing		SD	*VRN-H2*	**vrn**-**H2**	Yes	0.50 days
Arta × Keel^10^	Field, winter sowing		LD	*VRN-H2*	**vrn**-**H2**	Yes	3.70 days
Plaisant × (Candela × 915006)^11^	Controlled conditions	Null	LD	*VRN-H2*	**vrn**-**H2**	Yes	2.40 days
*GWAS*							
HEB-25^12^	Field, spring sowing		LD	*VRN-H2*	**vrn**-**H2**	Yes	2.20 days
HEB-25^13^	Field, winter sowing		LD	*VRN-H2*	**vrn**-**H2**	Yes	1.50 days
HEB-25^14^	Field, spring sowing		LD	*VRN-H2*	**vrn**-**H2**	Yes	1.20 days

Surveys where associations between flowering time and the *VRN-H2* locus region have been detected are reported. It includes linkage mapping studies performed in biparental populations, as well as genome wide association analyses

^a^Environmental conditions, ^b^vernalization treatment (*w* weeks), ^c^photoperiod length (*LD* long days, *SD* short days), ^d^*VRN-H2* alleles, ^e^*VRN-H1* segregation state in the population, and ^f^*VRN-H2* additive effect were collected from the original sources. ^d^Alleles contributing to earliness are highlighted in bold

^1^Laurie et al. (1995), ^2^Karsai et al. (2005), ^3^Karsai et al. (2006), ^4^Karsai et al. (2008), ^5^Cuesta-Marcos et al. (2008b), ^6^Cuesta-Marcos et al. (2008a), ^7^Wang et al. (2010), ^8^Tondelli et al. (2014), ^9^Sameri et al. (2011), ^10^Rollins et al. (2013), ^11^Malosetti et al. (2011), ^12^Maurer et al. (2015), ^13^Saade et al. (2016), ^14^Herzig et al. (2018)

In addition, *VRN-H2* exerts pleiotropic effects on several developmental and agronomic traits. As expected, when vernalization cannot be completed timely, the presence of *VRN-H2* is deleterious for grain yield and yield components (Rollins et al. 2013). However, positive effects of the presence allele have also been reported. Lines with this allele showed more reproductive tillers, greater thousand grain weight (TGW) and grain yield, when fully vernalized (Karsai et al. 2006). This interesting finding should be confirmed in field trials with appropriate plant materials. Some evidence of field effects of *VRN-H2* on spring barleys is provided by Wang et al. (2010). Unique introgressions carrying *VRN-H2* showed delayed flowering (Table 2), reduced height, lodging severity and TGW, but an enhanced value in ears per square meter, harvest index and yield.

There is a particularly rich stream of experimental evidence for the pleiotropic effects of *VRN-H2* on multiple traits coming from the study of NAM populations. Besides lengthening of the stem elongation phase, shortening of the ripening phase, and the corresponding delay in flowering time (Table 2), wild barley alleles at *VRN-H2* (presence) were associated with reductions in plant height (Maurer et al. 2016; Nice et al. 2017; Herzig et al. 2018), particularly under high ambient temperature and salt stress (Saade et al. 2016).

In wheat, the *VRN-2* locus encodes two tandemly repeated *ZCCT* genes (Yan et al. 2004b). Deletions or recessive *vrn-2* loss-of-function alleles result in spring growth habit in both diploid and tetraploid wheat (Yan et al. 2004a; Distelfeld et al. 2009b). However, the combination of mutations in all three *VRN-2* homeologues, that would give rise to spring growth habit in hexaploid wheat, has not been observed in nature (Kippes et al. 2016). Apparently, there is no natural variation for this gene in the A and D subgenomes. Natural variation in gene copy number has been revealed for the *VRN-B2* locus, which also shows a stronger effect on vernalization requirement than other homeologues (*VRN-B2* > *VRN-D2*) (Distelfeld et al. 2009b; Kippes et al. 2016). *VRN-2* variation in wheat does not have the same clear-cut effect on growth habit as it has in barley, probably due to the complexity of polyploidy gene effect compensations. Variation at this locus could be used to expand allelic diversity for heading time and to broaden the adaptation of polyploid wheat (Kippes et al. 2016).

In summary, the epistatic interaction between *VRN-H2* and *VRN-H1* is the main factor controlling vernalization response in barley. *VRN-H2* repressing effect depends on the length of low-temperature exposure and photoperiod regime. Its effect on flowering is mostly visible in spring-sown trials or in insufficiently vernalized plants followed by long photoperiods. Additionally, *VRN-H2* exerts pleiotropic effects on agronomic traits such as height or grain yield components. This was proven in winter barleys under incomplete vernalization and deserves further investigation in spring barleys.

### *VRN-H3*

*VRN-H3* (*HvFT1*), on 7HS, is the barley orthologue of the *Arabidopsis FLOWERING LOCUS T* gene (Yan et al. 2006; Faure et al. 2007; Kikuchi et al. 2009), the main integrator of the photoperiod and vernalization signals leading to the transition from vegetative to reproductive state of the apical meristem. Its expression requires induction by long days, and increased transcript levels correlate with earlier flowering times (Turner et al. 2005; Yan et al. 2006). Mulki and Von Korff (2016) hypothesized that once the vernalization requirements are satisfied, *PPD-H1* and *HvCO1*/*CO2* up-regulate *VRN-H3*, inducing flowering under long-day conditions. On the other hand, the photoperiod insensitive *ppd-H1* allele, typical of spring types, has been associated with lower transcript levels of *VRN-H3* and delayed flowering under long days compared with the sensitive *PPD-H1* allele (Turner et al. 2005; Hemming et al. 2008).

*FT* encodes a mobile protein (florigen) produced in the leaves, then transported to the apices, where it triggers flowering (Corbesier et al. 2007; Li and Dubcovsky 2008). The induction of flowering is the result of complex interactions occurring in the shoot apical meristem (SAM). At the SAM, the FT protein interacts with the *bZIP* transcription factor FD to activate expression of the floral meristem identity genes *AP1* in *Arabidopsis* (Abe et al. 2005; Wigge et al. 2005), and *VRN-1* in wheat (Li and Dubcovsky 2008). Later, the same authors demonstrated that FT, other FT-like proteins and different FD-like proteins could interact with multiple wheat and barley 14–3-3 proteins (Li et al. 2015).

The regulation of *VRN-H3* expression is affected by some known transcription factors, which can result in the occurrence of QTL interactions in studies with mapping populations. In *A. thaliana*, Tiwari et al. (2010) described that the flowering time regulator CO binds to the promoter of *FT*, via a unique cis-element. Although this tight relationship has not been described in barley, there is evidence of an enhanced *VRN-H3* expression caused by *HvCO2* (Mulki and von Korff 2016). Also, Deng et al. (2015) showed that the VERNALIZATION 1 protein binds to the promoter of *VRN-H3* in barley, up-regulating its expression.

Ample allelic variation at *VRN-H3* has been described, arising from sequence polymorphisms in the promoter and first intron (Yan et al. 2006; Hemming et al. 2008; Casas et al. 2011), and from copy number variation (Nitcher et al. 2013; Loscos et al. 2014). However, a clear, unique nomenclature for *VRN-H3* alleles gathering all these polymorphisms has not been developed. Therefore, we propose a new *VRN-H3* allele designation that defines alleles based on their promoter and intron haplotypes, and specifies the number of copies of *HvFT1*, the gene underlying *VRN-H3* (Table S1). We aim at introducing a unifying allele nomenclature to ease the knowledge transfer between breeders and plant scientists, and to be routinely used in future studies.

Several reports in different biparental populations have detected flowering time QTL on the *VRN-H3* region of chromosome 7H, representing all types of polymorphism at *VRN-H3* (Table 3). Studies involving large germplasm collections also detected an important association between *VRN-H3* and flowering time (Pasam et al. 2012; Alqudah et al. 2014; Sharma et al. 2020, and other references in Table 3). The *VRN-H3* region also presented the most significant association with flowering time in multi-parent advanced generation inter-cross (MAGIC) population studies (Sannemann et al., 2015; Afsharyan et al., 2020).

**Table 3:** Polymorphisms at *VRN-H3* and effects on flowering

	Differential polymorphism^a^			*VRN-H3* allele^b^		Additive effect (days)^c^	Interaction (days)^d^	
Population	P	I	CNV	Parent 1	Parent 2		*VRN-H1*	*vrn-H1*
*Biparental populations*								
*H. spontaneum* × BGS213^1^	Late *vs* early	TC *vs* AG	1 *vs* 4*	*vrn-H3d(1)*	**VRN-H3a(T)**	33.00		
Igri × BGS213^1^	Late *vs* early	TC *vs* AG	1 *vs* 4*	*vrn-H3d(1)*	**VRN-H3a(T)**	35.50		
IMC × BGS213^2^			1 *vs* 4*	*vrn-H3a(1)*	**VRN-H3a(T)**	41.50		
*H. spontaneum* × Morex^2^	Late *vs* early	TC *vs* AG		*vrn-H3d(1)*	*vrn-H3a(1)*	ns		
Hayakiso 2 × IMC^2^		TC *vs* AG		*vrn-H3c(1)*	*vrn-H3a(1)*	ns		
*H. spontaneum* × E878^2^	Late *vs* early			*vrn-H3d(1)*	**vrn-H3c(1)**		4.8	19.5
*H. spontaneum* × U672^2^	Late *vs* early			*vrn-H3d(1)*	**vrn-H3c(1)**		30.0	8.5
Hayakiso 2 × *H. spontaneum*^2^	early *vs* Late			*vrn-H3c(1)*	**vrn-H3d(1)**	7.00		
SBCC016 × Esterel^3^		AG *vs* TC		*vrn-H3b(1)*	**vrn-H3d(1)**	3.50		
Beatrix × SBCC145^4^	Late *vs* early			*vrn-H3d(1)*	**vrn-H3c(1)**	2.40		
Mogador × Beka^5, 6^			1 *vs* 2	*vrn-H3d(1)*	**vrn-H3d(2)**	1.10		
SBCC154 × Beatrix^6^		AG *vs* TC	4 *vs* 1	*vrn-H3b(4)*	**vrn-H3d(1)**	1.30		
Henni × Meltan^6, 7^	Late *vs* early			*vrn-H3d(3)*	**vrn-H3c(3)**	1.50		
Beka × Logan^8^	Late *vs* early		2 *vs* 1	*vrn-H3d(2)*	**vrn-H3c(1)**	1.30		
Steptoe × Morex^9^	Late *vs* early	TC *vs* AG		*vrn-H3d(1)*	**vrn-H3a(1)**	0.40		
*GWAS*								
140 winter landraces (SBCC)^3^	Late *vs* early	AG *vs* TC		AG intron	**TC intron**	3.50		
HEB-25^10^	Late *vs* early			*vrn-H3d(1)*	**vrn-H3c(1)**	2.10		
MAGIC ^11,12^	Late *vs* early	AG *vs* TC	?	AG intron	**TC intron**	5.80/−0.20		
AB-NAM^13^	?	?	?	wild	**Rasmusson**	0.70		
Phenology diversity panel^14, 15^	?	AG *vs* TC	?	AG intron	**TC intron**	0.10		

Surveys where associations between heading time and the *VRN-H3* locus region were detected are reported. It includes linkage mapping studies performed in biparental populations segregating for *VRN-H3*, and genome wide association analyses

^a^Type/s of polymorphism differencing the parents (*P* promoter, *I* intron and *CNV* copy number variation). Contrasting haplotypes for each differential polymorphism are shown. *For CNV, the asterisk indicates the unique feature of having a single copy of the promoter and several copies of the transcribed region. ^b^*VRN-H3* alleles arise from the combination of polymorphisms at the P and I, and from CNV, as reported in Table S1. Alleles contributing to earliness are highlighted in bold. ^c^*VRN-H3* additive effects were collected from the original sources (*ns* nonsignificant effect). The populations cited were phenotyped under field conditions except for those from references ^1^ and ^2^, which were phenotyped under LD conditions and nonvernalizing temperatures. ^d^The effect of the interaction with *VRN-H1* alleles is presented (*VRN-H1*: spring allele, *vrn-H1*: winter allele)

^1^Yan et al. (2006), ^2^Nitcher et al. (2013), ^3^Casas et al. (2011), ^4^Ponce-Molina et al. (2012), ^5^Cuesta-Marcos et al. (2008b), ^6^Loscos et al. (2014), ^7^Borras-Gelonch et al. (2010), ^8^Casas et al. (2020), ^9^Borras-Gelonch et al. (2012), ^10^Maurer et al. (2015), ^11^Sanneman et al. (2015), ^12^Afsharyan et al. (2020), ^13^Nice et al. (2017), ^14^He et al. (2019), ^15^Hill et al. (2019)

Multiple copies of *VRN-H3* have only been detected in spring and facultative genotypes lacking *VRN-H2* (Loscos et al. 2014). If the VRN2 protein interacts directly with the mechanism of promotion of *VRN-H3* (Li et al. 2011), it could be hypothesized that *VRN-H3* CNV has not been found in winter cultivars because the VRN2 protein produced would not be able to repress several copies of *VRN-H3*. Nitcher et al. (2013) showed that the presence of multiple copies of certain spring barley *VRN-H3* allele was associated with earlier up-regulation of *VRN-H3*, earlier flowering, and an overriding effect of the vernalization mechanism, later confirmed by Cuesta-Marcos et al. (2015). This overriding effect of vernalization came only from the *VRN-H3* allele present in the barley genetic stock BGS213 (derived from the Finnish cultivar Tammi), and not from other CNV alleles. This allele, hereafter named *VRN-H3a(T)* (T from Tammi) had the unique feature of having a single copy of the promoter and several copies of the transcribed region (Nitcher et al. 2013). *VRN-H3a(T)* is dominant over the rest of *VRN-H3* alleles described (Yan et al. 2006). It was reportedly found only in spring cultivars originating from regions of extremely high latitude or high altitude, where it seems to be particularly beneficial (Takahashi and Yasuda 1971). Loscos et al. (2014) found no clear relation between CNV, gene expression and flowering time for other alleles present in spring/facultative barleys.

Regarding sequence variation, Yan et al. (2006) described two promoter haplotypes characterized by seven linked SNPs and two InDels (insertion/deletion) in the first 550 bp upstream of the start codon (InDel 1-InDel 2: insertion-deletion *vs* deletion-insertion), and two first intron haplotypes characterized by two linked polymorphisms (AG *vs* TC). They reported a strong phenotypic effect associated only with the first intron polymorphism. While the AG allele was initially featured as conferring earliness, this was later corrected when more data from mapping populations were available (Casas et al. 2011), and now the TC allele is currently acknowledged as the “early” allele. Another source of confusion could stem from the strand that the *VRN-H3* intron SNP markers from the Illumina 9 K and 50 K chips (12_30894 and 12_30895) are called. These markers are targeting the bottom strand, where the early TC allele would be read as AG. Casas et al. (2011) analyzed natural variation for promoter and intron 1 haplotypes in a landrace collection of predominantly winter barleys (SBCC). In this latter survey, four main *VRN-H3* haplotypes (*vrn-H3a-d*, Table S1) were associated with flowering time differences, which geographical distribution strongly correlated with latitude. The intron TC haplotype showed significantly earlier flowering (6–8 days) than the AG haplotype. The prevalence of the early allele (TC) in southern Spanish barley landraces suggests an adaptation role for the *VRN-H3* gene. The presence of the TC allele may be convenient for plants growing in mid-spring in Mediterranean climates, to escape from rapidly rising temperatures and the risk of terminal drought and heat stress. Conversely, barley landraces from Northern Europe carry predominantly the AG haplotype (Aslan et al. 2015), suggesting that the geographical distribution of *VRN-H3* allelic diversity plays a role in adaptation. In addition, Casas et al. (2011) found that polymorphisms at the *VRN-H3* promoter also contributed to the gene effect on flowering time. The deletion in InDel 1 (early promoter hereafter) was associated with earlier heading (2–3 days) than the insertion (late promoter hereafter) in autumn sowings. The landraces carrying the combination of the early promoter with the TC intron were associated with the earliest flowering (Casas et al. 2011; Ponce-Molina et al. 2012). This class, *vrn-H3c*, actually represents a distinct allele with more polymorphisms in the promoter compared to other classes, as later confirmed by Nitcher et al. (2013). Likewise, in the population Beka (*vrn-H3d(2)*) x Logan (*vrn-H3c(1)*), the Logan *VRN-H3* allele was associated with earlier flowering (Casas et al. 2020). Borràs-Gelonch et al. (2012) detected a QTL for flowering time in the population Steptoe (*vrn-H3d(1)*) x Morex (*vrn-H3a(1)*) close to the *VRN-H3* region. This QTL was only significant in some environments, indicating the expected balancing effect of the two polymorphisms, intron and promoter.

Under spring-sown field conditions, a strong epistatic interaction was found between the regions corresponding to *VRN-H3* and *PPD-H1*, with a strong flowering delay caused by the combination of the insensitive *ppd-H1* allele and the late *vrn-H3a* allele (Afsharyan et al. 2020). Ponce-Molina et al. (2012) also found this interaction in an autumn sowing. As in the study of Afsharyan et al. (2020), the allelic effect at *VRN-H3* was maximized in the presence of the insensitive allele *ppd-H1.* However, under autumn sowing conditions, the photoperiod insensitive allele accelerated flowering time. Finally, there is a recent report by Bi et al. (2019), suggesting that *HvCEN* genetically interacts with *VRN-H3* to modulate floral development.

In addition to flowering time, pleiotropic effects of *VRN-H3* have been reported on duration of developmental phases, plant height, low-temperature tolerance, yield and yield-related traits (Wang et al. 2010; Chutimanitsakun et al. 2013; Mansour et al. 2014; Maurer et al. 2016; Nice et al. 2017; Sharma et al. 2018). These effects probably stem from the relationship between earliness and yield. A rich source of experimental evidence of the pleiotropic effects of *VRN-H3* comes from the study of barley populations derived from a spring elite cultivar x wild accession(s) cross (Wang et al. 2010; Maurer et al. 2015; Nice et al. 2017). Wild alleles at the *VRN-H3* region have been associated with delayed development, including shooting, stem elongation, heading and maturity phases (Maurer et al. 2016), increased height (Maurer et al. 2016; Nice et al. 2017), and reduced performance in harvest index and yield (Wang et al. 2010; Sharma et al. 2018).

The richness of polymorphisms and flowering time effects found at *VRN-H3* may provide breeders with additional genetic variability to fine-tune plant development to local environmental conditions.

As in barley, *VRN-3* plays a central role in the integration of signals from vernalization and photoperiod pathways in wheat, with a similar mechanism. This gene presents homeologue copies in subgenomes A, B and D. Sequence variation has been reported for *VRN-A3* (non-synonymous substitution in exon 3) and *VRN-D3* (InDel in exon 3), both having small effects on flowering time (Bonnin et al. 2008). A natural insertion of a retrotransposon element in the promoter region of *VRN-B3* is associated with a stronger early flowering effect under long-day photoperiods (Nitcher et al. 2014), and induced mutations at *VRN-A3* and *VRN-B3* also affect flowering time (Lv et al. 2014). This richness of genetic variation makes this gene one of the main breeding targets to adjust wheat heading time to changing environments.

Summarizing, this key flowering promoter integrates the vernalization and photoperiod pathways. Its expression requires long days and fulfilment of vernalization requirements in winter barleys. Recent studies have revealed ample allelic variation, likely indicating different regulation mechanisms, associated with phenotypic effects. Further additional variation for barley flowering is provided by epistatic interactions with *PPD-H1* and *VRN-H1*. The allelic richness at this locus and its central role in the flowering pathways suggest that it plays a key role in adaptation and agronomic fitness, and offers a large catalogue of options for plant breeders.

## Photoperiod response

Barley is a long-day plant, with genetic sensitivities to both long and short photoperiod (Laurie et al. 1995). Two main genes, *PPD-H1* and *PPD-H2*, have been proposed as the main drivers of these responses.

### *PPD-H1*

The *PPD-H1* locus has been identified as the major determinant of long photoperiod response in barley (Turner et al. 2005). Both wild barley and landraces from southwest Asia, southern Europe, and the Mediterranean basin carry a dominant allele, which induces an early occurrence of flowering under increasing day length in spring. Spring landraces from central and northern Europe carry a recessive photoperiod-insensitive *ppd-H1* allele, which confers delayed flowering and maturity under long days (Turner et al. 2005; Hemming et al. 2008; Jones et al. 2008). The emergence of the nonresponsive *ppd-H1* allele, in combination with other mutations at different genes, clearly favored the expansion of barley production to higher latitudes (von Bothmer and Komatsuda 2011), by extending the period of vegetative growth of spring-sown plants, thus allowing higher accumulation of biomass, potentially supporting higher yields.

The *PPD-H1* locus encodes a *PSEUDO-RESPONSE REGULATOR* (*HvPRR37*) gene, orthologous to the *Arabidopsis* gene *PRR7*, and maps to the short arm of chromosome 2H. *HvPRR37* is part of the plant circadian clock and its activity causes an increased expression of *VRN-H3*, the main promoter of flowering, when photoperiods rise above 12 h (Turner et al. 2005; Campoli et al. 2012b). On the one hand, *PPD-H1* acts in parallel to *HvCO1* (Campoli et al. 2012a; Shaw et al. 2020). After vernalization, *PPD-H1* and *HvCO1*/*CO2* up-regulate *VRN-H3*, inducing flowering under long-day conditions (Mulki and von Korff 2016). On the other hand, mutations at evening complex genes *HvELF3* and *HvLUX1*, and *HvPHYC* modulate the expression of *PPD-H1.* Mutations in any of these genes result in a day-neutral up-regulation of *VRN-H3* and early flowering (Zakhrabekova et al. 2012; Faure et al. 2012; Nishida et al. 2013a; Campoli et al. 2013; Pankin et al. 2014). Turner et al. (2005) identified a single nucleotide polymorphism (G/T) at the *PPD-H1* locus (Table S1), leading to a change of amino acid in the CCT-domain, as potentially responsible for long photoperiod insensitivity, which has been confirmed recently (Sharma et al. 2020).

Polymorphisms at this gene abound, and its phylogeny has been well studied (Russell et al. 2016; Sharma et al. 2020). However, the phenotypic effects rarely indicate the presence of more than the two functionally distinct alleles described above, the sensitive (*PPD-H1*) and the insensitive (*ppd-H1*) ones. Some studies hint at the presence of alleles that are functionally different from those two (Hemshrot et al. 2019; Bustos‐Korts et al. 2019). On the one hand, several private alleles were found in Asian barleys conferring both positive and negative effects, which are not due to the same causative variant for European barley flowering time variation (Hemshrot et al. 2019). On the other hand, from the eight *PPD-H1* haplotypes described by Bustos‐Korts et al*.* (2019) in a global barley panel, haplotype g, classified as photoperiod-sensitive, accelerated flowering both under short and long-day conditions, indicating a response different from that typical of a photoperiod-responsive allele.

Several association-based studies involving wide germplasm collections have identified *PPD-H1* as a major player responsible for flowering time variation (Jones et al., 2008; Russell et al., 2016; He et al., 2019 and references in Table 4). Moreover, several of these studies showed a clear latitude-dependent geographical distribution of the two main *PPD-H1* alleles, with the nonresponsive (or, better, less responsive) form predominant in the North (Jones et al. 2008; Lister et al. 2009; Russell et al. 2016; Bustos‐Korts et al. 2019). It is well established that *PPD-H1* shows stronger effects on heading date under long photoperiod conditions (e.g., winter or spring sowings), with the sensitive allele conferring earliness (Laurie et al. 1994, 1995; Boyd et al. 2003; Cuesta-Marcos et al. 2008a; Maurer et al. 2015; Boudiar et al. 2016; Mikołajczak et al. 2016). However, a crossover interaction between *PPD-H1* and the environment has been reported independently for several barley populations (Table 4), namely Dicktoo × Morex (Pan et al. 1994), Steptoe × Morex (Borràs-Gelonch et al. 2012), SBCC145 x Beatrix (Ponce-Molina et al. 2012), and Plaisant x Orria (Mansour et al. 2014). In these studies, a significant QTL by environment interaction for heading date was detected in the region of *PPD-H1*, with the sign and magnitude of the *PPD-H1* effect varying depending on the environment. The insensitive *ppd-H1* allele conferred earliness in autumn sowings in the Mediterranean region, in which most of the growing season occurred under short days. On the contrary, in winter or spring sowings, or autumn sowings with a larger proportion of the growing season under long days, the sensitive *PPD-H1* allele conferred earliness. The delaying effect of the sensitive *PPD-H1* allele in early flowering trials is small, but it is credible, given its consistency across four different populations. Field-based GWAS studies confirm this interaction (Bustos‐Korts et al*.*, 2019). Likewise, Wiegmann et al. (2019) found a latitude and photoperiod-dependent *PPD-H1* effect. The wild (sensitive) allele of *PPD-H1* accelerated flowering time only in locations exceeding 12 h photoperiod during the shooting phase, and the effect was higher with increasing latitude. In addition, the interaction was evident when comparing results of the HEB-25 population from spring-sown German trial, in which the sensitive *PPD-H1* allele reduced time to heading by 9.5 days (Maurer et al. 2015) with autumn-sown Israel (Merchuk-Ovnat et al. 2018) and Dubai trials (Saade et al. 2016), where the sensitive *PPD-H1* allele increased heading time by 6.7 and 2 days, respectively.

**Table 4:** Interaction of *PPD-H1* effect and environment on flowering time

Population	Environment (sowing/photoperiod)^a^	Location^b^	Latitude^c^	*PPD-H1* allele^d^		Additive effect^e^	Sowing date	Heading date	DTH^f^	ZD^g^
				Parent 1	Parent 2					
*Biparental populations*										
Dicktoo × Morex^1^	Phytotron, 8 h	Martonvasar (HU)	47° 18′ N	*PPD-H1*	*ppd-H1*	ns			105.0	
Dicktoo x Morex^1^	Phytotron, 16 h	Martonvasar (HU)	47° 18′ N	**PPD-H1**	*ppd-H1*	7.80			45.0	
Dicktoo × Morex^1^	Greenhouse	Oregon (US)	44° 24′ N	**PPD-H1**	*ppd-H1*	16.10			52.5	
Igri × Triumph^2^	Field, autumn	Norwich (UK)	52° 38′ N	**PPD-H1**	*ppd-H1*	6.00				Z55
Igri × Triumph^2^	Field, spring	Norwich (UK)	52° 38′ N	**PPD-H1**	*ppd-H1*	10.80				Z55
Igri x Triumph^2^	Greenhouse, 10 h	Norwich (UK)	52° 38′ N	*PPD-H1*	*ppd-H1*	ns				Z55
Igri × Triumph^2^	Greenhouse, 18 h	Norwich (UK)	52° 38′ N	**PPD-H1**	*ppd-H1*	10.00				Z55
Dicktoo × Morex^3^	Phytotron, 16 h, 18 °C	Martonvasar (HU)	47° 18′ N	**PPD-H1**	*ppd-H1*	12.00			44.0	
Dicktoo × Morex^3^	Phytotron, 16 h, 18/16 °C	Martonvasar (HU)	47° 18′ N	**PPD-H1**	*ppd-H1*	13.00			74.0	
Dicktoo × Morex^3^	Phytotron, 24 h, 18 °C	Martonvasar (HU)	47° 18′ N	**PPD-H1**	*ppd-H1*	9.00			42.0	
17 interconected pop.^4^	Field, autumn	Lupinén (ES)	42° 10′ N	*PPD-H1*	*ppd-H1*	ns	Nov-08	Apr-19	110.0	Z49
17 interconected pop.^4^	Field, autumn	Zuera (ES)	42° 09′ N	*PPD-H1*	*ppd-H1*	ns	Nov-15	Apr-19	110.0	Z49
17 interconected pop.^4^	Field, winter	Alerre (ES)	41° 00′ N	**PPD-H1**	*ppd-H1*	2.50	Jan-28	May-19	140.0	Z49
17 interconected pop.^4^	Field, winter	Zuera (ES)	42° 09′ N	**PPD-H1**	*ppd-H1*	3.10	Jan-22	May-19	140.0	Z49
Azumamugi × KNG^5^	Phytotron, 12 h	Tsukuba (JP)	36° 01′ N	**PPD-H1**	*ppd-H1*	19.11			189.0	Z49
Azumamugi × KNG^5^	Field, autumn	Tsukuba (JP)	36° 01′ N	*PPD-H1*	*ppd-H1*	ns			185.0	Z58
Azumamugi x KNG^5^	Field, spring	Tsukuba (JP)	36° 01′ N	*PPD-H1*	*ppd-H1*	ns			54.0	Z58
SBCC145 × Beatrix^6^	Field, autumn	Zaragoza (ES)	41° 43′ N	*PPD-H1*	**ppd-H1**	−1.09	Oct-29	Apr-11	102.0	Z49
SBCC145 × Beatrix^6^	Field, winter	Zaragoza (ES)	41° 43′ N	**PPD-H1**	*ppd-H1*	3.32	Feb-08	May-14	135.2	Z49
Steptoe x Morex^7^	Field, autumn	Lleida (ES)	41° 37′ N	*PPD-H1*	**ppd-H1**	−0.82	Nov-21	Apr-25		Z55
Steptoe × Morex^7^	Field, autumn	Gimenells (ES)	41° 38′ N	*PPD-H1*	**ppd-H1**	−0.60	Nov-30	Apr-20		Z55
Steptoe × Morex^7^	Field, autumn, ext. PD	Lleida (ES)	41° 37′ N	**PPD-H1**	*ppd-H1*	0.59	Nov-21	Apr-19		Z55
Steptoe × Morex^7^	Field, winter	Gimenells (ES)	41° 38′ N	**PPD-H1**	*ppd-H1*	2.43	Feb-26	May-09		Z55
Steptoe × Morex^7^	Greenhouse, spring	Lleida (ES)	41° 37′ N	**PPD-H1**	*ppd-H1*	3.66	Mar-23	May-20		Z55
Plaisant × Orria^8^	Field, autumn	Gimenells (ES)	41° 39′ N	*PPD-H1*	**ppd-H1**	−0.30	Dec-01	Apr-16	107.7	Z49
Plaisant × Orria^8^	Field, autumn	Bell-lloc (ES)	41° 37′ N	*PPD-H1*	**ppd-H1**	−0.40	Nov-02	Apr-21	112.9	Z49
Plaisant × Orria^8^	Field, autumn	Sádaba (ES)	42° 17′ N	**PPD-H1**	*ppd-H1*	1.30	Nov-22	May-01	122.3	Z49
Plaisant × Orria^8^	Field, autumn	Sádaba (ES)	42° 17′ N	**PPD-H1**	*ppd-H1*	0.50	Nov-26	Apr-25	116.5	Z49
Plaisant × Orria^8^	Field, winter	Fiorenzuola(IT)	44° 56′ N	**PPD-H1**	*ppd-H1*	2.70	Mar-01	May-23	144.9	Z49
SBCC073 × Orria^9^	Field, autumn	Zuera (ES)	42° 09′ N	**PPD-H1**	*ppd-H1*	1.50	Nov-11	Mar-02	111.0	Z49
Cam × Maresi^10^	Field, spring	Cerekwica (PL)	52° 31′ N	**PPD-H1**	*ppd-H1*	2.79	Apr-10	May-31	51.4	Z51
Cam × Lubuski^10^	Field, spring	Cerekwica (PL)	52° 31′ N	**PPD-H1**	*ppd-H1*	2.42	Apr-10	May-30	50.7	Z51
Harmal × Georgie^10^	Field, spring	Cerekwica (PL)	52° 31′ N	**PPD-H1**	*ppd-H1*	1.68	Apr-09	May-26	47.9	Z51
*GWAS*										
HEB-25^11^	Field, autumn	Rehovot (IL)	31° 54′ N	*PPD-H1*	**ppd-H1**	−3.40	Dec-03	Mar-16	102.5	Z49
HEB-25^12^	Field, autumn	Dubai (AE)	25° 05′ N	*PPD-H1*	**ppd-H1**	−1.00	Dec-08	Feb-26	82.6	Z55
HEB-25^13^	Field, spring	Halle (DE)	51° 29′ N	**PPD-H1**	*ppd-H1*	4.75	Mar/Apr		68.1	Z49
HEB-25^14^	Field, spring	Dundee (UK)	56° 28′ N	**PPD-H1**	*ppd-H1*	3.00	Apr		78.4	Z49
HEB-25^14^	Field, spring	Halle (DE)	51° 29′ N	**PPD-H1**	*ppd-H1*	3.90	Mar		69.4	Z49
WHEALBI subset^15^	Field, autumn	Martonvasar (HU)	47° 17′ N	*PPD-H1*	**ppd-H1**	−3.20	Oct-20	May-03	195.1	Z55
WHEALBI subset^15^	Field, autumn	Fiorenzuola (IT)	44° 53′ N	*PPD-H1*	**ppd-H1**	−1.90	Oct-27	May-02	187.2	Z55
WHEALBI subset^15^	Field, autumn	Dundee (UK)	56° 30′ N	*PPD-H1*	**ppd-H1**	−1.70	Oct-29	Jun-07	222.3	Z55
WHEALBI subset^15^	Field, winter	Martonvasar (HU)	47° 17′ N	**PPD-H1**	*ppd-H1*	1.30	Mar-11	May-22	72.7	Z55
WHEALBI subset^15^	Field, winter	Dundee (UK)	56° 30′ N	**PPD-H1**	*ppd-H1*	2.50	Mar-03	May-25	83.1	Z55
HEB-YIELD^16^	Field, autumn, 11 h	Dubai (AE)	25° 05′ N	*PPD-H1*	*ppd-H1*	ns	Dec		89.4	Z49
HEB-YIELD^16^	Field, autumn, 10.5 h	Adelaide (AU)	35° 19′ S	*PPD-H1*	*ppd-H1*	ns	May/Jun		124.5	Z49
HEB-YIELD^16^	Field, spring, 16 h	Dundee (UK)	56° 28′ N	**PPD-H1**	*ppd-H1*	3.80	Mar/Apr		84.6	Z49
HEB-YIELD^16^	Field, spring, 15 h	Halle (DE)	51° 29′ N	**PPD-H1**	*ppd-H1*	4.40	Mar		66.1	Z49
HEB-YIELD^16^	Field, winter, 12 h	Al-Karak (JO)	31° 16′ N	**PPD-H1**	*ppd-H1*	3.40	Dec/Jan		108.9	Z49
AB-NAM^17^	Field, spring	Minnesota (US)	47° 46′ N	**PPD-H1**	*ppd-H1*	3.00	May-08	Jun-28	51.2	Z55
BRIDG6^18^	Field, spring	Minnesota (US)	47° 46′ N	**PPD-H1**	*ppd-H1*	4.50	May-04	Jun-24	50.8	Z58
MAGIC^19^	Field, spring	Bonn (DE)	50° 36′ N	**PPD-H1**	*ppd-H1*	0.36	Apr-07	Jun-14	68.5	Z49

Surveys where associations between flowering time and the *PPD-H1* locus region were detected are reported. It includes linkage mapping studies performed in biparental populations segregating for *PPD-H1*, and genome wide association analyses

^a^Environmental conditions (*ext. PD* extended photoperiod), ^b^location, ^c^latitude, ^d^*PPD-H1* alleles and ^e^additive effects in days were collected from the original sources. ^d^Alleles contributing to earliness are highlighted in bold. ^e^Additive effect on heading time in days (substitution of one sensitive *PPD-H1* allele by one insensitive *ppd-H1* allele). Negative sign indicates that *ppd-H1* promotes flowering, positive sign indicates that *ppd-H1* delays flowering (*ns* nonsignificant effect). ^f^Days to heading from sowing (underlined) or from January 1^st^; ^g^Zadoks stage, developmental phase measured as flowering time in each experiment

^1^Pan et al. (1994), ^2^Laurie et al. (1995), ^3^Karsai et al. (2008), ^4^Cuesta-Marcos et al. (2008a), ^5^Sameri et al. (2011), ^6^Ponce-Molina et al. (2012), ^7^Borràs-Gelonch et al. (2012), ^8^Mansour et al. (2014), ^9^Boudiar et al. (2016), ^10^Mikołajczak et al. (2016), ^11^Merchuk-Ovnat et al. (2018), ^12^Saade et al. (2016), ^13^Maurer et al. (2015), ^14^Herzig et al. (2018), ^15^Bustos-Korts et al. (2019), ^16^Wiegmann et al. (2019), ^17^Nice et al. (2017), ^18^Hemshrot et al. (2019), ^19^Afsharyan et al. (2020)

**Fig. 2:**
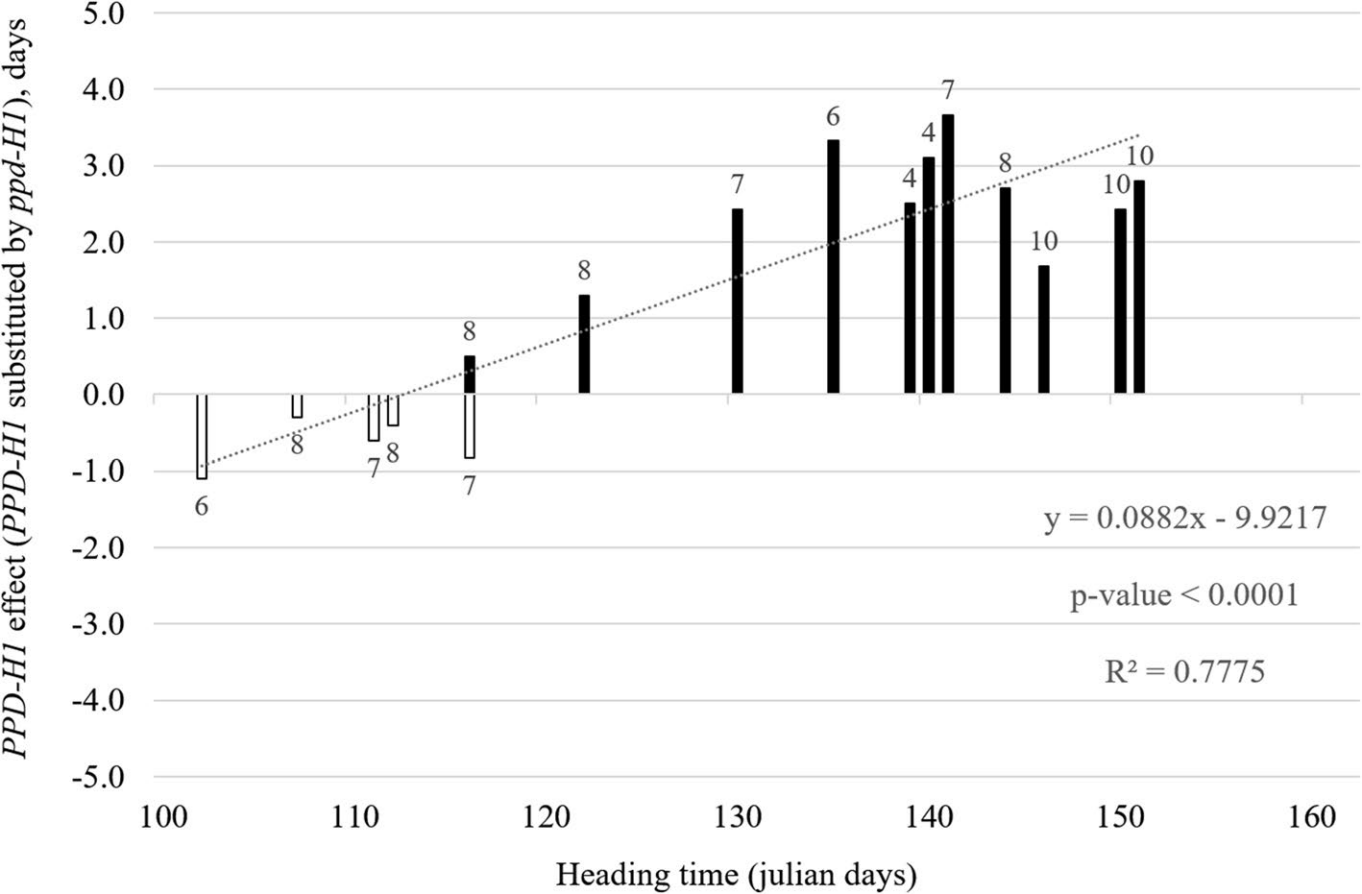
Interaction of the effect of *PPD-H1* with environment. Additive effect of *PPD-H1* detected in several barley mapping populations plotted according to average field heading date. The additive effect of *PPD-H1* is calculated as the average effect in flowering time when one sensitive *PPD-H1* allele is substituted by one insensitive *ppd-H1* allele. White bars indicate earliness conferred by the insensitive *ppd-H1* allele. Black bars indicate earliness conferred by the sensitive *PPD-H1* allele. Numbers above or below bars indicate the study from which the data was obtained. The correspondence between numbers and references is located in the footnote of Table 4. The regression line (dotted line), the linear equation, the coefficient of determination (R^2^), and the significance of the regression analysis are shown

Figure 2 summarizes the effects of QTL at the *PPD-H1* region found in biparental populations. A change in the direction of the *PPD-H1* effect occurs at approximately 112 Julian days. This could be valid for a certain range of temperatures and latitudes. All the studies summarized in the graph come from latitudes between 40 and 50°N because Julian dates were available only for those. The crossover point may vary for trials at lower or higher latitudes, and different temperatures and pace of thermal time accumulation. Therefore, it is not surprising that this *PPD-H1* × environment interaction was also observed in Scotland for two trials sown in autumn and spring, flowering in May–June (Bustos‐Korts et al. 2019). For photoperiod-sensitive genotypes (*PPD-H1* allele) to benefit from the accelerating effect of long days, the rhythm of accumulation of growing degree-days has to be such that the occurrence of the inducing photoperiod coincides with the leaf initiation phase, and this depends not only on the latitude but also on the local climate. Studying the causes of the *PPD-H1* × environment interaction could shed further light on the mechanism of barley response to photoperiod.

*PPD-H1* is a central gene in the photoperiod developmental pathway, and is rich in interactions with genes upstream and downstream. The interaction between *PPD-H1* and *VRN-H3* (or, at least, QTL with those underlying genes as candidates) has strong experimental backing, as explained in the *VRN-H3* section. von Korff et al. (2010) found an interaction, between *PPD-H1* and *HvCO2*. The *PPD-H1* sensitive allele accelerated flowering only in presence of an exotic allele at *HvCO2*, while it did not show an effect in combination with the most common allele at this locus. In the latter work, an interaction between *PPD-H1* and *VRN-H2* was also found. The wild (sensitive) *PPD-H1* allele only promoted flowering in a genetic background lacking *VRN-H2*. Mulki and von Korff (2016) found again a link between these two genes, whose nature depended on whether it takes place before or after vernalization (reviewed in ‘*VRN-H2*′ section). Ejaz and von Korff (2017) demonstrated that under high ambient temperature, flowering time is controlled by interactions between *PPD-H1* and *VRN-H1*. Only in the background of a spring *VRN-H1* allele or after up-regulation of *vrn-H1* by vernalization, the wild-type *PPD-H1* allele is capable of accelerating early reproductive development under high ambient temperatures.

In addition to heading time, pleiotropic effects of *PPD-H1* have been reported on many relevant agronomic and morphological traits, like plant height, leaf size, root growth or yield components (Laurie et al. 1994; Karsai et al. 1999; von Korff et al. 2006; Bauer et al. 2009; Wang et al. 2010; Mansour et al. 2014; Maurer et al. 2016; Digel et al. 2016; Alqudah et al. 2018; Abdel-Ghani et al. 2019; Wiegmann et al. 2019).

*PPD-H1* seems to act in a location-specific manner on yield-related traits, mostly (but not only) in connection with earliness. At those locations where earliness is beneficial (e.g., early plants can escape higher temperatures and terminal drought at the end of the growing season), the responsive/sensitive allele of *PPD-H1* has been associated with an increase in yield. The yield effect may be explained through pleiotropic effects of the responsive *PPD-H1* allele, which shortens the overall growing season, increases the period of grain filling and increases grain size. On the other hand, at those locations where lateness is preferable to achieve higher yields, the nonresponsive *ppd-H1* allele has been associated with increases in yield-related traits (Wiegmann et al. 2019). However, the current long growing season characteristic of Northern Europe might increasingly change towards Mediterranean conditions as a consequence of climate change, and the ecological advantages of *ppd-H1* could thus disappear in some regions (Herzig et al. 2018).

In this regard, *PPD-H1* not only perceives day length but also seems to interact with temperature to regulate plant development in barley (Borràs-Gelonch et al. 2012; Hemming et al. 2012; Ford et al. 2016; Ejaz and von Korff 2017; Herzig et al. 2018). Ejaz and von Korff (2017) found that the sensitive allele of *PPD-H1* accelerated floral development and maintained the seed number under high ambient temperatures, whereas the insensitive *ppd-H1* allele delayed floral development and reduced the number of florets and seeds per spike. In addition, Gol et al. (2021) recently showed that variation at *PPD-H1* interacts with drought to control flowering time and yield. Lines with a photoperiod-responsive *PPD-H1* allele showed higher trait stability in response to drought. Considering the upcoming environmental conditions, the sensitive *PPD-H1* allele may gain more importance in spring barleys in latitudes North of the Mediterranean region, although possible negative effects on tillering should be compensated.

The wheat homologues of *PPD-H1* are *PPD-A1*, *PPD-B1* and *PPD-D1* located on chromosomes 2A, 2B and 2D, respectively (Laurie 1997; Beales et al. 2007). Wild-type alleles (‘b’ suffix, e.g., *PPD-A1b*; Mcintosh et al. 2003) are associated with day-length sensitivity, whereas mutations in *PPD-1* genes (‘a’ suffix) result in photoperiod insensitivity. Apparently, there are differences between wheat and barley photoperiod responses. In wheat, genotypes carrying the photoperiod-insensitive allele flower rapidly regardless of whether they are exposed to short or long-day conditions, whereas in barley, the consensus names as “insensitive alleles” those that delay flowering under long days (Turner et al. 2005). Photoperiod-sensitive alleles in wheat and barley substantially delay heading under short days. It is worth noting that both barley and wheat will accelerate flowering to some extent under long days, even in genotypes with the alleles of *PPD-1* that confer strong day-length insensitivity (Hyles et al. 2020). As in barley, wheat *PPD-1* interacts with temperature to accelerate flowering (Hemming et al. 2012).

The molecular mechanisms underlying photoperiod sensitivity may differ between the two species. Allelic diversity in *PPD-1* results from deletions or a transposon insertion in the promoter, and from copy number variation (*PPD-B1*) (Wilhelm et al. 2009; Bentley et al. 2011; Díaz et al. 2012; Bentley et al. 2013; Nishida et al. 2013b; Zhang et al. 2015a, b; Würschum et al. 2019). The homeologue in D subgenome is the major factor affecting flowering time in hexaploid wheat germplasm (Kiss et al. 2014; Langer et al. 2014; Würschum et al. 2018). Moreover, there is a dosage effect, lines combining photoperiod-insensitive alleles on two or three genomes had enhanced earliness (Shaw et al. 2012; Ochagavía et al. 2017). In wheat, higher expression of some *PPD-1* alleles confers earliness (Shaw et al. 2012; Kiss et al. 2017), something not seen in barley. This is consistent with the type of polymorphisms found, in regulatory regions or CNV for wheat, and in the coding region (CCT domain) of barley. Plant breeders using genome editing may use the knowledge of these different mechanisms underlying photoperiod response in barley and wheat in the future.

In brief, *PPD-H1* is the major gene responsible for photoperiod response in barley. Two main functional alleles have been reported, although recent evidence suggests that there might be more. Finding out the effects of new *PPD-H1* alleles should be prioritized in barley research. The marked latitudinal distribution of the two main alleles and their effects on relevant agronomic traits supports its strong adaptive role. *PPD-H1* effect on flowering time shows a crossover interaction with the environment. The sensitive allele of *PPD-H1* confers earliness under long days. However, a delay of flowering by the responsive allele under short days has been consistently reported. *PPD-H1* interacts with temperature and drought to regulate plant development and acts in a location-specific manner on yield-related traits. New conditions arising from climate change may call for redefining the agronomic fitness of *PPD-H1* alleles for each region.

### *PPD-H2*

The *PPD-H2* locus was first identified as a modifier of flowering time, manifested in response to short days (Laurie et al. 1995). *HvFT3*, another *FT*-*like* member of the PEBP family, is the candidate gene underlying this locus and was mapped to chromosome 1H (Faure et al. 2007; Kikuchi et al. 2009). Attending to its phenotypic effect, only two allelic variants are known: a dominant one, with a functional copy of the gene, and a recessive allele, with most of the gene missing and non-functional (Kikuchi et al. 2009) (Table S1). The dominant, functional allele is prevalent in spring barley and winter barley landraces and cultivars from southern Europe (< 44°N). Its effect is more complex than initially thought and promotes flowering in short-day conditions, or even long-day conditions when vernalization requirements have not been fully satisfied (Casao et al. 2011a, c). The non-functional recessive allele is mainly found in central and northern European winter barley (Kikuchi et al. 2009). This uneven distribution across geographic and germplasm divides points at a relevant adaptation role for *PPD-H2*, confirmed by environmental association studies which identified *PPD-H2* as a divergent selection signature between groups of barley landraces (Contreras‐Moreira et al. 2019; Lei et al. 2019).

The non-functional *ppd-H2* allele originated pre-domestication (Cockram et al. 2011). Within winter barleys, the mutated *ppd-H2* allele was favored in northern latitudes, characterized by longer seasons where sufficient vernalization is ensured, and early transition to reproductive growth would expose the plants at a high risk of winterkill. The null, late-flowering allele helps autumn-sown cultivars maintain the vegetative growth phase longer (Pan et al. 1994), perhaps through maintaining the expression of genes that confer tolerance to low temperature (Fowler et al. 2001). However, the dominant ancestral *PPD-H2* allele was conserved in southern latitudes characterized by higher temperatures, where it might help to induce flowering when the vernalization requirement has not been satisfied in full. In spring cultivars, *PPD-H2* can facilitate flowering and ensure timely completion of such a short vital cycle, particularly in combination with “late” alleles at other loci (for instance, *HvCEN*), providing an adequate balance of duration of phenological phases to optimize yield. Therefore, *PPD-H2* likely plays an important adaptive role in spring barleys, and also in winter barleys, where it seems to act as a compensatory mechanism to accelerate flowering and ensure it occurs at the optimal time (Casao et al. 2011c).

During the last years, the view of *PPD-H2* as the “short photoperiod” gene has given way to a more complex regulation and function. *PPD-H2* is actually expressed both under short and long days, although its expression is more pronounced under short-day conditions (Faure et al. 2007; Kikuchi et al. 2009; Casao et al. 2011a). Kikuchi et al. (2009) reported that overexpression of *PPD-H2* resulted in early heading. However, its effect on heading time was weaker than that of *VRN-H3*, suggesting an indirect role of *PPD-H2* in the promotion of floral transition (Kikuchi et al. 2009). Recently, the findings of Mulki et al. (2018) supported this role: overexpression of *PPD-H2* accelerated the initiation of spikelet primordia and the early reproductive development, independently of photoperiod length. However, overexpression of *PPD-H2* did not accelerate floral development, and inflorescences aborted under short days, suggesting that *PPD-H2* controls spikelet initiation but not floral development, which necessitates of additional factors.

Regarding the regulation of *PPD-H2* expression, it has been hypothesized that it is repressed by *VRN-H2* (Casao et al. 2011a). In winter genotypes, with an active *VRN-H2* gene, its transcripts must be absent or clearly receding (either because lack of induction under short days, or repression by expression of *VRN-H1*) for *PPD-H2* to be expressed (Casao et al. 2011a), and this happens only after some cold exposure, and increasingly with plant age (Monteagudo et al. 2019b). In this last study, it was demonstrated that *PPD-H2* expression in a winter genotype is not induced merely by short days. In spring genotypes, most of them involving the deletion of *VRN-H2*, *PPD-H2* is expressed without restriction, even under long days, although to a lesser extent than under short days. Casao et al. (2011a) demonstrated that the up-regulation of the *PPD-H2* transcript correlated with increased levels of *VRN-H1* and *VRN-H3* expression. In contrast, Mulki et al. (2018) reported that overexpression of *PPD-H2* was associated with a strong up-regulation of *VRN-H1*, but not *VRN-H3* in the leaf under both photoperiods. Additionally, *PPD-H2* upregulated the expression of barley row-type genes *VRS4*, *VRS1*, and *INT*-C, which suggested that *FT*-like genes may control spike architecture in addition to modulating developmental timing (Mulki et al. 2018).

*PPD-H2* was identified originally as a major heading time QTL in winter × spring barley crosses under short photoperiod conditions (Laurie et al., 1995 and references in Table 5). The dominant *PPD-H2* allele was associated with earliness under early-sown field and short-day glasshouse experiments. Several association-based studies involving large germplasm collections have also identified *PPD-H2* as a major flowering time QTL in a worldwide survey of barley germplasm (Alqudah et al., 2014 and references in Table 5). The largest effects on growth occurred until the stage of awn tipping, although, in the *PPD-H1* group, it was still visible until anther extrusion. Under controlled conditions, an apparent substitution of the vernalization requirement by exposure to short photoperiod conditions in winter genotypes was observed (Laurie et al. 1995; Cuesta-Marcos et al. 2008b), phenomenon that was previously named “short-day vernalization” (Roberts et al. 1988). Mulki et al. (2018) concluded that *PPD-H2* does not only counteract the repressive effect of the vernalization pathway, but also induces early reproductive development of winter barley under short-day conditions. *PPD-H2* seems to play a dual role in the induction of flowering by promoting spikelet initiation under short days and by reducing the requirement for vernalization under long days, as *PPD-H2* seemed to cause a down-regulation of *VRN-H2* in the absence of vernalization. Therefore, *PPD-H2* constitutes an adaptive mechanism to mild winters (at least milder than central-European ones).

**Table 5:** Polymorphisms at *PPD-H2* and effects on flowering time in barley mapping populations.

Population	Environment/conditions^a^	*PPD-H2* allele^b^		Additive effect (days)^c^
		Parent 1	Parent 2	
*Biparental populations*				
Igri × Triumph^1^	Field, autumn sowing	*ppd-H2*	***PPD-H2***	3.40
Igri × Triumph^1^	Field, spring sowing	*ppd-H2*	**PPD-H2**	0.70
Mogador × Beka^2^	Field, autumn sowing	*ppd-H2*	**PPD-H2**	2.00
Mogador × Beka^2^	Field, winter sowing	*ppd-H2*	**PPD-H2**	0.90
Mogador × Beka^2^	Field, spring sowing	*ppd-H2*	*PPD-H2*	ns
17 interconected populations^3^	Field, autumn sowing	*ppd-H2*	**PPD-H2**	1.40
17 interconected populations^3^	Field, winter sowing	*ppd-H2*	*PPD-H2*	ns
Steptoe × Morex^4^	Field, autumn sowing	*ppd-H2*	**PPD-H2**	2.10
Steptoe × Morex^4^	Field, winter sowing	*ppd-H2*	**PPD-H2**	0.60
Steptoe × Morex^4^	Field, spring sowing	*ppd-H2*	*PPD-H2*	ns
Plaisant × (Candela × 915006)^5^	Field, autumn sowing	*ppd-H2*	**PPD-H2**	1.60
Azumamugi × KNG^6^	Field, autumn sowing	*ppd-H2*	**PPD-H2**	3.30
Cierzo × SBCC073^7^	Field, autumn sowing	*ppd-H2*	**PPD-H2**	0.60
Cierzo × SBCC042^7^	Field, autumn sowing	*ppd-H2*	**PPD-H2**	1.30
*GWAS*				
HEB-25^8^	Field, autumn sowing	Wild	**PPD-H2**	1.00
Spring world collection^9^	Field, spring sowing	*ppd-H2*	**PPD-H2**	2.50
WHEALBI subset^10^	Field, autumn sowing	*ppd-H2*	**PPD-H2**	1.00
WHEALBI subset^10^	Field, winter sowing	*ppd-H2*	**PPD-H2**	0.70

Surveys where associations between flowering time and the *PPD-H2* locus region were detected are reported. It includes linkage mapping studies performed in biparental populations segregating for *PPD-H2*, and genome wide association analyses

^a^Environmental conditions, ^b^*PPD-H2* alleles, and ^c^additive effect were collected from the original sources (*ns* nonsignificant effect). ^b^Alleles contributing to earliness are highlighted in bold

^1^Laurie et al. (1995), ^2^Cuesta-Marcos et al. (2008b), ^3^Cuesta-Marcos et al. (2008a), ^4^Borras-Gèlonch et al. (2012), ^5^Malosetti et al. (2011), ^6^Sameri et al. (2011), ^7^Monteagudo et al. (2019a), ^8^Saade et al. (2016), ^9^Pasam et al. (2012), ^10^Bustos-Korts et al. (2019)

For this reason, the *PPD-H2* effect is most influential in Mediterranean latitudes, where autumn-sown cultivars experience short photoperiods during most of the growing season. In early-sowings, it has been identified as one of the two largest effect QTL affecting flowering time, together with *HvCEN* (Boyd et al. 2003; Cuesta-Marcos et al. 2008a, b; Malosetti et al. 2011) (Table 5). However, a lower magnitude but significant effect of *PPD-H2* was also detected in vernalized plants grown under long photoperiods (Cuesta-Marcos et al. 2008a, b). This quantitative QTLxE interaction at the *PPD-H2* region is clearly exemplified in the study by Borràs-Gelonch et al. (2012). The effect of *PPD-H2* was gradual, larger in autumn sowings than in winter sowings, while it was absent in spring sowings. In addition, this survey reported that the effect of *PPD-H2* was higher on the leaf and spikelet initiation phase than in the stem elongation phase, which is in agreement with Mulki et al. (2018) findings. Karsai et al. (2008) also identified the *PPD-H2* locus as a significant determinant of flowering time under long photoperiods, but it presented an interaction with temperature, as its effect was only seen when synchronous photo- and thermocycles were applied, and not under constant temperature. *PPD-H2* effect also depended on the allelic configurations at *PPD-H1* and *VRN-H1*, with largest effect on winter-type haplotypes, particularly with the insensitive *ppd-H1* allele. The effect of this gene has been observed outside the temperate regions. A flowering time QTL with a strong effect under short photoperiod was observed in subtropical latitudes (Sameri et al. 2011).

The adaptive role of *PPD-H2* is confirmed by its influence on key agronomic traits, as mentioned recurrently in the literature. Cuesta-Marcos et al. (2009) reported that *PPD-H2* affected grain yield indirectly, through flowering date, under Mediterranean conditions. As expected, its effect was dependent on the environment. The dominant allele was significantly superior in environments where earliness conferred a yield advantage (e.g., terminal stress), whereas the opposite was true for the recessive allele. Mansour et al. (2018) confirmed the beneficial effect of dominant *PPD-H2* on yield of winter types evaluated in Egyptian conditions, by hastening development under short days. Two populations developed from the cross of a Spanish landrace and the elite cultivar Cierzo shared a QTL hotspot on the *PPD-H2* region (Monteagudo et al. 2019a). The QTL contributed to variation in flowering time, TGW, soil coverage, and hectoliter weight. In both populations, flowering was accelerated by the dominant *PPD-H2* allele, which also increased TGW. In the same region, better soil coverage was contributed by the landrace SBCC042 but coincident with a lower hectoliter weight. On the negative side, a dominant *PPD-H2* seems to reduce frost tolerance in winter and facultative genotypes (Cuesta-Marcos et al. 2015; Rizza et al. 2016). In the HEB-25 population, Sharma et al. (2018) found pleiotropic effects of the *PPD-H2* region (though co-location was not fully certain) on grain area, grain length, and grain roundness.

Orthologues of *PPD-H2* have been identified in the A, B and D genomes of hexaploid and tetraploid wheat (Halliwell et al. 2016). As in barley, these genes are upregulated under short photoperiods (Halliwell et al. 2016; Zikhali et al. 2017). The whole deletion of *TaFT3-B1* gene was associated with late flowering, paralleling the results of genotypes carrying the recessive *ppd-H2* in barley. Wheat *TaFT3-B1* gene, however, presents more allelic variation, with CNV and a non-synonymous substitution (also associated with late flowering), besides the presence/absence alleles similar to barley (Zikhali et al. 2017).

In summary, *PPD-H2* induces early reproductive development under non-inductive conditions. Its prevailing effect is found under short photoperiod conditions, e.g., autumn sowings. However, its effect has also been detected under long days, when vernalization is incomplete, e.g., winter or spring sowings. The functional allele (*PPD-H2*) predominates in spring and southern winter barley, whereas the non-functional (*ppd-H2*) does it in central and northern winter barley. This distribution hints at an adaptive role in spring and winter barleys, where it could promote spikelet initiation, and ensuring that flowering occurs at the optimal time. Expression of *PPD-H2* in winter barleys has a complex regulation. It has an antagonistic relationship with *VRN-H2*, but it also needs some time of cold exposure. This gene presents large pleiotropic effects on agronomic traits beyond flowering time. Its presence/absence should be a question to be addressed in any breeding program aiming at winter genotypes for temperate regions.

### Earliness *per se* genes

Besides vernalization and photoperiod response genes, the rest of QTL detected affecting flowering time were classically grouped under the generic term “earliness *per se*” or “*eps*” (Laurie et al. 1995). Over the last years, several of these genes have been cloned. Some of them actually have major effects on phenology, and the most important one affecting adaptation is *HvCEN*.

#### *HvCEN*

The *eam6* or *eps2* locus, located in the centromeric region of chromosome 2H (Laurie et al. 1995), has been identified as an orthologue of the *Antirrhinum CENTRORADIALIS* gene, designated *HvCEN* (Comadran et al. 2012)*.* This gene is orthologous to *Arabidopsis TFL1*, a member of the *FT*-like gene family, but in contrast to *FT*, encodes a flowering repressor.

A worldwide survey of *HvCEN* genetic variation across wild and cultivated barley detected 14 SNPs that defined 13 haplotypes, 3 prevalent (HI, HII and HIII), shared between wild and domesticated barleys, and several minor ones. Phylogenetic analyses indicate that HIII was selected from wild barley and became fixed in European spring barley cultivars, whereas HII predominates in wild barleys from the eastern Mediterranean and in cultivated winter barleys. Therefore, *HvCEN* has been identified as a relevant contributor to the expansion of barley cultivation into diverse habitats, and as a signature of divergent selection between spring and winter cultivars (Comadran et al. 2012).

A single SNP in the last exon of *HvCEN* encodes a Pro135Ala amino acid change that differentiates barleys with the two main growth habits. Haplotypes HI and HIII harbor the mutation encoding Ala135, and have been associated with later flowering than haplotype HII-Pro135 (Comadran et al. 2012) (Table S1). HI and HIII differ in non-coding regions, two SNPs in intron 2 and one in the 3′UTR. Each allele would be beneficial under different environmental conditions, as follows: in Mediterranean rain-fed conditions (hot and dry summers), the winter Pro135-encoding allele would accelerate development, providing a mechanism to escape terminal drought. On the contrary, in long cool seasons, the spring Ala135-encoding allele would confer an advantage because it would delay flowering, lengthening grain filling under well-watered conditions (Comadran et al. 2012). Exome sequencing of geographically diverse barley landraces and wild relatives indicated that the Pro135Ala mutation in *HvCEN* was the most associated with latitude of all tested flowering- associated gene SNPs (Russell et al. 2016), supporting the hypothesis that *HvCEN* natural variation played an important role in environmental adaptation of cultivated barley.

It is not clear, whether the phenotypic effects of this gene stem only from modifications in the proteins or if gene expression levels are also involved. Comadran et al. (2012) pointed out that protein sequence changes were sufficient to justify the flowering phenotypes, but they also found a constitutive higher expression of HIII (late) than HII (early) alleles, which could also affect the phenotype. Bi et al. (2019) also found expression differences of *HvCEN* haplotypes. They found differential tissue expression of *HvCEN* HI (Bowman) and HIII (Bonus), which are identical for the proposed diagnostic polymorphism (Ala135). These haplotypes also present differences in regulatory regions, which could underlie the distinct expression patterns. Recently, the barley pan-genome has revealed an inversion tightly linked to the *HvCEN* region, which was possibly selected during the barley geographical range expansion and seems exclusive of the HIII carriers. Further research is required to determine whether this inversion has direct functional consequences, for instance, by modulating *HvCEN* expression (Jayakodi et al. 2020).

Flowering time QTL at the *HvCEN* region were first detected by Laurie et al. (1995). They found a QTL with the largest effect on flowering time, both in autumn and spring sowings, identified as *eps2*. The lines with the spring allele (Triumph, HIII) were consistently associated with later flowering than the lines with the winter allele (Igri, HII), with an effect between 2.5 days in the autumn sowing to 3.2 days in the spring one (Table 6). Since then, a good number of linkage studies in barley populations, tested on a wide variety of environments, have detected flowering time QTL on the *HvCEN* region (Boyd et al. 2003; Pillen et al. 2003, 2004; Sameri and Komatsuda 2004; Horsley et al. 2006; von Korff et al. 2006, 2008; Castro et al. 2008 and references in Table 6), and the same has been reported in GWAS studies (Pasam et al. 2012; Muñoz-Amatriaín et al. 2014; Alqudah et al. 2014; Maurer et al. 2016; Russell et al. 2016 and references in Table 6).

**Table 6:** Polymorphisms at *HvCEN* and effects on flowering time in barley mapping populations

Population	Environment/conditions^a^	*HvCEN* haplotype^b^		Additive effect (days)^c^	Interaction *VRN-H1*^d^
		Parent 1	Parent 2		
*Biparental populations*					
Triumph × Igri^1^	Field, autumn sowing	III	**II**	2.50	
Triumph × Igri^1^	Field, spring sowing	III	**II**	3.20	
Harrington × Morex^2^	Field, autumn sowing	III	**I**		
Beka × Logan^3^	Field, autumn sowing	III	**I**	2.20	
Beka × Logan^3^	Field, spring sowing	III	**I**	2.50	
KNG × Azumamugi^4^	Field, autumn sowing	III	**II**	2.40	
KNG × Azumamugi^4^	Field, spring sowing	III	**II**	1.70	
Beka × Mogador^5^	Field, autumn sowing	III	**II**	2.70	*vrn-H1*
Beka × Mogador^5^	Field, winter sowing	III	**II**	2.30	*vrn-H1*
Beka × Mogador^5^	Field, spring sowing	III	**II**	4.70	*vrn-H1*
17 interconected pop.^6^	Field, autumn sowing	III	**I, II**	1.80	
17 interconected pop.^6^	Field, winter sowing	III	**I, II**	1.40	
Beatrix × SBCC145^7^	Field, autumn sowing	III	**VI**	3.10	
Beatrix × SBCC145^7^	Field, winter sowing	III	**VI**	2.90	
Steptoe × Morex^8^	Field, autumn sowing	III	**I**	2.50	
Steptoe × Morex^8^	Field, winter sowing	III	**I**	2.50	
Tremois × Nure^9^	Field, autumn sowing	III	**II**	2.50	
Tremois × Nure^9^	Field, winter sowing	III	**II**	2.30	
Tremois × Nure^9^	Field, spring sowing	III	**II**	2.70	
Baronesse × Full Pint^10^	Field, autumn sowing	III	**II**	1.80	
Baronesse × Full Pint^10^	Field, winter sowing	III	**II**	2.00	
Orria × SBCC073^11^	Field, autumn sowing	I	**II**	1.30	*VRN-H1-4*
*GWAS*					
HEB-25^12^	Field, autumn sowing	III	**Wild**	3.00	
HEB-25^13^	Field, autumn sowing	III	**Wild**	3.80	
HEB-25^14^	Field, spring sowing	III	**wild**	1.50	
HEB-25^15^	Field, spring sowing	III	**Wild**	1.20	
AB-NAM^16^	Field, spring sowing	Wild	**Rasmusson**	0.50	
WHEALBI subset^17^	Field, autumn sowing	Late (III, I)	**Early (II)**	5.00	
WHEALBI subset^17^	Field, winter sowing	Late (III, I)	**Early (II)**	2.50	
MABDE^18^	Field	III, I	**II**	3.70	
Uruguay panel^19^	Field, winter sowing	III	**II**	2.40	
Phenology diversity panel^20, 21^	Field, autumn sowing	III	**II**	1.70	

Surveys where associations between flowering time and the *HvCEN* locus region were detected are reported. It includes linkage mapping studies performed in biparental populations segregating for *HvCEN*, and genome wide association analyses

^a^Environmental conditions, ^b^*HvCEN* alleles, and ^c^additive effect were collected from the original sources. ^b^Alleles contributing to earliness are highlighted in bold. ^d^The effect of the interaction with *VRN-H1* alleles is presented (*VRN-H1-4*: reduced vernalization requirement allele, *vrn-H1*: winter allele)

^1^Laurie et al. (1995), ^2^Márquez-Cedillo et al. (2001), ^3^Casas et al. (2020), ^4^Sameri and Komatsuda (2004), ^5^Cuesta-Marcos et al. (2008b), ^6^Cuesta-Marcos et al. (2008a), ^7^Ponce-Molina et al. (2012), ^8^Borras-Gelonch et al. (2012), ^9^Tondelli et al. (2014), ^10^Castro el at (2017), ^11^Boudiar et al. (2016), ^12^Saade et al. (2016), ^13^Merchuk-Ovnat et al. (2018), ^14^Maurer et al. (2015), ^15^Herzig et al. (2018), ^16^Nice et al. (2017), ^17^Bustos-Korts et al. (2019), ^18^Comadran et al. (2011), ^19^Locatelli et al. (2013), ^20^He et al. (2019), ^21^Hill et al. (2019)

Two field experiments carried out with winter x spring populations, with polymorphisms similar to Igri x Triumph, detected QTL at the *HvCEN* region as the most important for flowering time variation in Southern Europe, for sowing dates ranging from autumn to spring (Cuesta-Marcos et al. 2008b; Tondelli et al. 2014). Certainly, the most conspicuous effect of *HvCEN* on flowering time has been identified in autumn sowings in Mediterranean latitudes/climates (Moralejo et al. 2004; Cuesta-Marcos et al. 2008a, b; Ponce-Molina et al. 2012), including Australian environments, where it was identified as the major contributor to heading date variation for several mapping populations (Boyd et al. 2003). All these studies detected effects due to the polymorphism between HII and HIII. Fewer studies focused on polymorphisms involving HI. A phenotypic difference attributable to haplotypes I and III was found in crosses involving North-American germplasm (Marquez-Cedillo et al. 2001; Moralejo et al. 2004; Borràs-Gelonch et al. 2012; Casas et al. 2020). In all of them, HI conferred earliness over HIII (Table 6). The difference in flowering time between HI and HIII found in these studies indicates that the amino acid change is not solely responsible for phenotypic variation. Considering the differential tissue expression of HI and HIII found by Bi et al. (2019), a polymorphism in the 3′ region of *HvCEN* could be relevant for regulation of gene expression with potential phenotypic effects.

The question of which is the developmental phase most affected by this gene is not settled yet in the literature. Boyd et al. (2003) hypothesized that this locus was associated with variation in the timing of floral initiation (i.e., duration of the vegetative phase). However, other studies have reported a *HvCEN* effect mainly in the length of the stem elongation phase (Borràs-Gelonch et al. 2012; Castro et al. 2017). In GWAS studies, it was reported that all developmental phases were shortened, except ripening phase, when the spring Barke (HIII) elite alleles were substituted with the exotic alleles at the *HvCEN* region (Maurer et al., 2015). Moreover, Herzig et al. (2018) suggested that one of the wild alleles tested in their study offered a considerable lengthening of the ripening phase in Northern European environments.

**Fig. 3:**
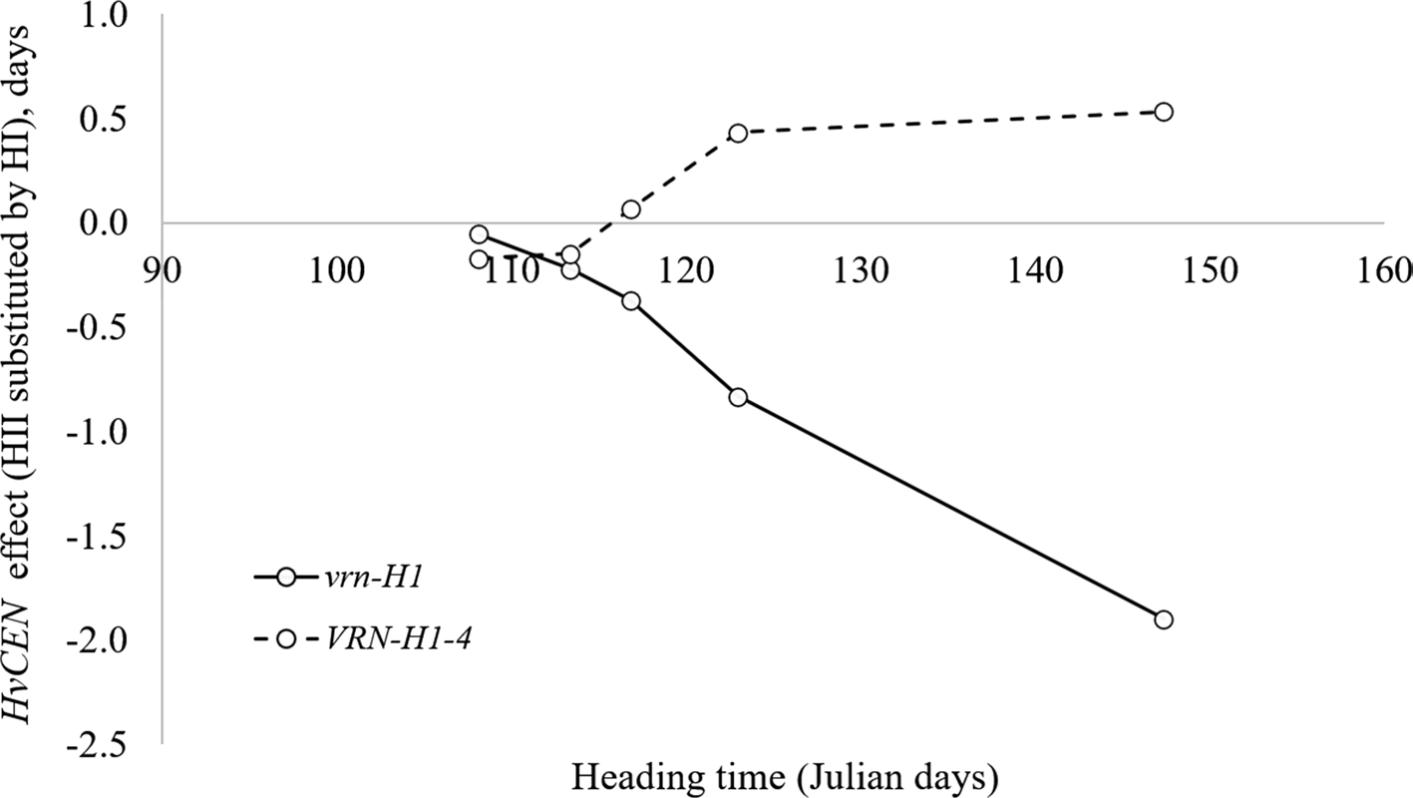
Interaction effect between *HvCEN* and *VRN-H1* on heading time in the Orria x Plaisant population, estimated in 5 field trials. The x-axis represents the average Julian days to flowering per trial. The additive effect of *HvCEN* represented in the y-axis is calculated as the average effect in flowering time when one HII allele is substituted by one HI allele. The solid line represents the *HvCEN* effect across heading times in the presence of the winter *vrn-H1* allele. The dashed line represents the *HvCEN* effect across heading times in the presence of the reduced vernalization requirement *VRN-H1-4* allele. The difference in flowering time between homozygous genotypes (HI-HII) would be double of the additive effect shown. Data reanalyzed from Mansour et al. (2014)

*HvCEN* seems to be in a central position of flowering pathways, due to its involvement in many interactions with other known flowering time genes. Laurie et al. (1995) detected a small but significant interaction between *HvCEN* and *PPD-H1,* and *HvCEN* and *VRN-H3* in a spring-sown trial. Besides, they found a contrasting effect of the *HvCEN* x *VRN-H1* interaction between autumn and spring sowings. Later, other studies hinted at the presence of an interaction between *HvCEN* and *VRN-H1* (Table 6)*.* Cuesta-Marcos et al. (2008b) only found this interaction in spring sowings. Lines carrying the HIII allele headed significantly later only when the winter allele of *VRN-H1* was present. Mansour et al. (2014) also identified an interaction between these genes in the mapping population Plaisant (HII, *vrn-H1*) x Orria (HI, *VRN-H1*-*4*), as shown in Fig. 3, although it was not published in that article. HI allele conferred earliness compared to HII, only in the presence of a winter allele at *VRN-H1*, with differences increasing in parallel to the average Julian date of flowering of the field trials (solid line, Fig. 3). The winter *vrn-H1* allele delayed heading time in all trials, but especially in the March-sown trial that experienced high temperatures. However, the delay in flowering time associated with that allele was reduced in the presence of the HI at *HvCEN*, only in late flowering trials. These results could indicate that the effect of *HvCEN* is influenced by day length, or some other environmental feature correlated with it.

It is possible that other genes take part in this interaction but, given the population size, no definitive conclusions could be drawn. In the population SBCC073 (HII, *VRN-H1-4*) x Orria (HI, *VRN-H1-4*) studied by Boudiar et al. (2016), the Orria allele (HI) at *HvCEN* was associated with late flowering time, which agrees with the late effect of the HI in the presence of the *VRN-H1-4* allele found in Orria x Plaisant. In both studies (Mansour et al. 2014; Boudiar et al. 2016), there were significant grain yield QTL interactions involving the *HvCEN* region, indicating its potential interest for plant breeding.

*HvCEN* also presented significant interactions with *VRN-H2*, in plants that had been vernalized and then grown under short photoperiod (Cuesta-Marcos et al. 2008b). Recently, the study of Bi et al. (2019) indicated that *HvCEN* interacts with *PPD-H2* to control spikelet initiation and with *VRN-H3* to repress floral development. Given the known relationship between *VRN-H2* and *PPD-H2*, these two findings could be connected. Finally, Casas et al. (2020) found several epistatic interactions involving *HvCEN*, *HvELF3*, *HvFD-like* and *VRN-H3*. In the triple interaction *HvELF3* x *HvCEN* x *VRN-H3*, *VRN-H3* had a large effect, particularly when all three alleles came from Logan, producing marked earliness. In the *HvELF3* x *HvCEN* x *HvFD-like* case, the effect of *HvFD-like* was overridden by the presence of Beka alleles at the other two QTL, resulting in late flowering.

Apparently, external cues affect the size of the effect of flowering time QTL coincident with *HvCEN*. van Eeuwijk et al. (2010) indicated that lower minimum temperatures during heading were associated with larger QTL effects in the population Steptoe x Morex, evaluated across North-America and Scotland locations.

Several pleiotropic effects have been detected for flowering time QTLs on the *HvCEN* region (Pillen et al. 2003, 2004; Moralejo et al. 2004; Horsley et al. 2006; Castro et al. 2008; Cuesta-Marcos et al. 2009; Borràs-Gelonch et al. 2012; Rollins et al. 2013; Tondelli et al. 2014; Mansour et al. 2014; Boudiar et al. 2016; Obsa et al. 2017). In most cases, they are a consequence of the large influence of earliness on other traits. The *HvCEN* region has been described as a hotspot for grain yield-related traits in several populations. A study with three interconnected genetic populations developed from the cross of Australian elite barley genotypes, confronting HII and HIV (deduced from authors’ data), detected a yield QTL on *HvCEN* region (Obsa et al. 2017), even though both haplotypes are Pro135. Haplotype II parents contributed the high-yield allele, with no relation to maturity. Yield and yield components (mostly, TGW) QTLs have been commonly found in populations tested in Mediterranean environments, confronting HII and HIII (Nure x Tremois, Tondelli et al., 2014; Beka x Mogador, Cuesta-Marcos et al., 2009). In another population, confronting HIII and HI (Beka x Logan), the *HvCEN* region harbored a QTL by environment (QTLxE) for TGW (Moralejo et al. 2004), with an increasingly larger favorable effect of the early allele (HI) on TGW the later the flowering date of the trial. Other studies have reported grain yield QTL on the *HvCEN* region presenting a crossover interaction between environments (Francia et al. 2011; Mansour et al. 2014; Boudiar et al. 2016). In populations confronting HI and HII (Orria x Plaisant, Mansour et al. 2014; SBCC073 x Orria, Boudiar et al. 2016), HII reduced yield in a late flowering spring-sown trial, while increasing it in an early flowering autumn-sown trial. All these relationships seemed a consequence of significant correlations between earliness and yield. This relationship was usually negative, although QTL x environment qualitative interactions were evident in some cases, depending on the sign of the earliness-yield relationship.

There is a large body of experimental evidence for the pleiotropic effects of *HvCEN* on multiple traits from the study of the HEB-25 NAM population (Maurer et al. 2016; Saade et al. 2016; Merchuk-Ovnat et al. 2018; Herzig et al. 2018; Pham et al. 2019). Briefly, their findings confirm the effects of *HvCEN* alleles studied in biparental populations, although it is not clear in some cases (Merchuk-Ovnat et al. 2018) if the effects detected are caused by *HvCEN* or by some closely linked gene. It is also worth mentioning that *HvCEN* was reported as the gene underlying yield-related traits QTL when this population was tested under field stress conditions. Both under drought (Merchuk-Ovnat et al. 2018; Pham et al. 2019) and salinity (Saade et al. 2016), *HvCEN* wild alleles offered better agronomic performance, under control and stress conditions, compared to the allele contributed by the cultivated parent (Barke, carrying HIII).

*HvCEN* is yet another example of the benefits of genetic diversity in adaptation. The stable effect observed across very different agrometeorological conditions revealed its more general role in wide adaptation, and this is further confirmed by the detection of a QTL for yield adaptability at the same genomic locus (Tondelli et al. 2014).

This gene has not been highlighted in wheat as underlying any QTL of agronomic relevance. A search of genomic databases using *HvCEN* as a template finds three hits, corresponding by sequence and position to two orthologues on chromosomes 2B and 2D, and a third orthologue with no position assigned, annotated as “Terminal flower 1”. There were very similar sequences in all six whole genome sequenced wheat varieties for two of these genes. In genome D, there was a predicted amino acid change differentiating them.

In summary, *HvCEN* contributes to the differentiation between spring and winter cultivars. Three main haplotypes have been described (I, II and III). The early haplotype II predominates in winter barleys, whereas the haplotype III does in spring types. These two haplotypes differ at a single amino acid (Pro135Ala), and show a clear latitudinal distribution, suggesting an adaptive role. Differences in flowering time between haplotypes I and III, however, may be regulatory. Although haplotype II has been classically acknowledged as the “early” allele, plants carrying haplotype I in some genetic backgrounds, have been associated with even earlier flowering. The *HvCEN* region is frequently identified as a hotspot of QTL, QTL × environment, and QTL x QTL effects on flowering, and on yield-related traits, suggesting a central position for *HvCEN* in the flowering pathways. These interactions should be investigated further for their potential application in breeding.

### Other genes affecting flowering time used in modern barley breeding

Besides vernalization and photoperiod pathways, circadian clock-related *earliness *per se genes have had a relevant role in the barley breeding history to expand the agroecological range of the crop even further (Faure et al. 2012). *HvCO1* and *HvCO2* are LD-flowering promoters modulated by circadian clock and day length (Griffiths et al. 2003; Campoli et al. 2012a; Mulki and von Korff 2016). In wheat, CO2 competes with VRN2 to bind the NF-Y proteins, in a mechanism to integrate environmental cues through regulation of *VRN-H3* (Li et al. 2011). *HvPHYC* is a phytochrome receptor, functioning as a red and far-red light sensor, key to entrain the circadian clock and perceive the photoperiod (Franklin and Quail 2010). *HvPHYC* is involved in a complex gene interaction network (Pankin et al. 2014; He et al. 2019). Interestingly, an early *PHYC-e* allele was selected in barley cultivars from Japan, where it may provide a selective advantage (Pankin et al. 2014). Several population analysis studies support the role of *HvPHYC* in flowering time variation (Mikołajczak et al. 2016; Ibrahim et al. 2018; Hu et al. 2019; Hill et al. 2019; Sato et al. 2020), as well as its effect in other agronomic traits, including yield (Tesso Obsa et al. 2016; Gong et al. 2016; Hill et al. 2019). In the same pathway, mutations at *HvELF3* (*Mat-a* or *eam8*) within recessive *ppd-H1* stocks, were identified as a major earliness factor facilitating adaptation of barley to very short seasons, at high latitudes (Zakhrabekova et al. 2012; Faure et al. 2012).

Recently, increasing attention is being paid to the Gibberellic acid (GA)-dependant pathway, as an important regulator of key development stages in short-day conditions (Pham et al. 2020). Genes within this pathway enabled the adaptation to modern agriculture, thereby have been widely used in barley improvement, as is the case of the dwarfing or semi-dwarfing genes *Slender 1* (*SLN1*) (Chandler et al. 2002), *breviaristatum-e* (*ari-e*) (Liu et al. 2014), and *semi-dwarf 1* (*sdw1*/*denso*) (Mickelson and Rasmusson 1994; Hellewell et al. 2000). The semi-dwarfing varieties have better lodging resistance, higher harvest index, and more efficient utilization of the environment (Milach and Federizzi 2001). The *sdw1*/*denso* gene, however, has been associated with deleterious effects such as late heading and maturity, decreased TGW, and decreased grain weight (Thomas et al. 1991, 1995; Mickelson and Rasmusson 1994; Powell et al. 1997; Hellewell et al. 2000; Jia et al. 2011). The combination of both semi-dwarfing genes, *sdw1* and *ari-e*, in lines of a recently developed MAGIC population suggest a compensation of flowering time between the two genes, further shortening plant height, and maintaining or slightly increasing grain yield (Dang et al. 2020).

## Concluding remarks

**Fig. 4:**
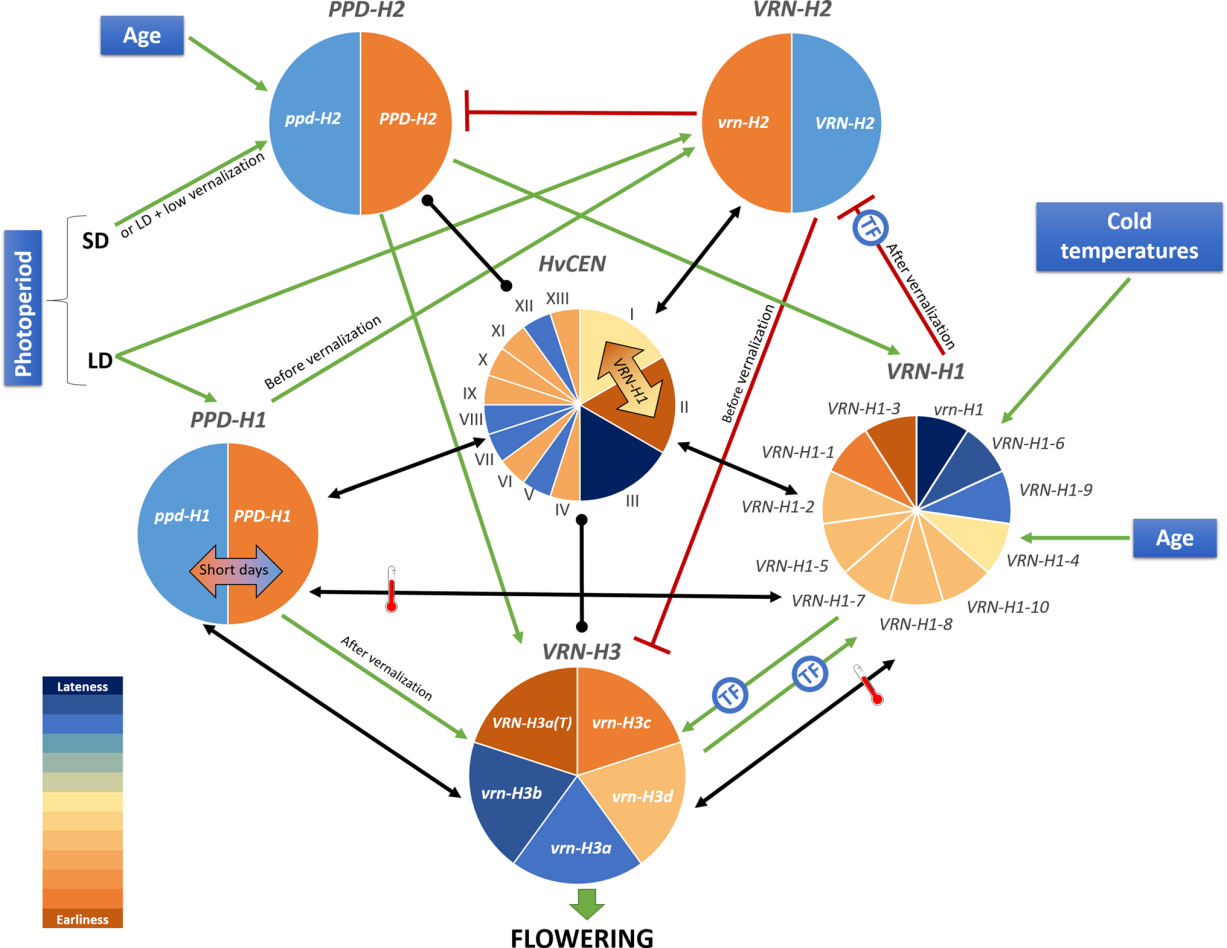
Allelic series, effects, interactions between genes and with the environment for six major flowering time genes of barley. Each gene is represented by a circle, sectors represent the alleles that have been reported with phenotypic effect for each gene. The scale of colors indicates the degree of promotion or repression for each allele, between brown (early) and blue (late). Blue boxes indicate external or internal cues. Green lines with arrows and red lines with blunt ends, respectively indicate positive and negative regulatory actions. Black lines indicate epistatic interactions detected in different types of studies: round, gene x gene interaction; arrowed, QTL x QTL interaction. The TF badge (transcription factor) indicates evidence for protein-DNA interaction. The thermometer icon indicates that the QTL x QTL interaction was observed under high temperature. The arrow within the *PPD-H1* circle indicates earliness conferred by the insensitive *ppd-H1* allele under short days, and the opposite under long days. The arrow within the *HvCEN* circle indicates a crossover interaction of the effect of haplotypes I and II dependent on the *VRN-H1* allele. *LD* long days, *SD* short days

This review summarizes the allelic variation, effects, interactions between genes and with the environment, for the six major flowering time players that have driven barley adaptation to diverse growing environments (Fig. 4). Considering the wide catalogue of alleles and effects described above, it seems clear that the flowering time genetic variation available to date is enough to tune-up phenology to different formats. However, more extreme ideotypes will be needed with the upcoming environmental conditions brought about by climate change. One potential source of flowering time variation could stem from CNV. Until recently, CNV in flowering time genes in barley had been described in *VRN-H2* (Dubcovsky et al. 2005) and *VRN-H3* (Nitcher et al. 2013; Loscos et al. 2014), whereas in wheat, it was detected in *VRN-A1*, *VRN-B2*, *PPD-B1*, and *TaFT3-B1* (Díaz et al. 2012; Würschum et al. 2015, 2018, 2019; Kippes et al. 2016; Zikhali et al. 2017). However, the recent publication of the barley pan-genome (Jayakodi et al. 2020) allows searching for new variation accessible to scientists and breeders. We identified several mutations responsible for amino acid changes within *ZCCT-Ha*, *ZCCT-Hb*, or *PPD-H1* genes, which could be further explored to assess possible phenotypic differences. Another source of plasticity in flowering behavior could derive from refining the phases that comprise the plant cycle. The duration of the vegetative and early reproductive phases determines the final number of spikelets, while the late reproductive phase (LRP) determines the number of fertile florets, thus the number of grains and potential yield (Alqudah and Schnurbusch 2014; Digel et al. 2015). The length of the preanthesis phenological phases is genetically controlled, and these developmental periods show different sensitivity to environmental stimulus (Slafer and Rawson 1994; Miralles and Richards 2000; González et al. 2002; Gol et al. 2017; Ochagavía et al. 2018). *VRN-H1*, *VRN-H2*, and *PPD-H2* have been associated mainly with the length of the vegetative and early reproductive phases. *VRN-H3*, *PPD-H1*, and *HvCEN*, however, seem to affect the length of the LRP. Breeders to fine-tune varietal vernalization needs to the target environments can use the gradual vernalization responses provided by the allelic series at *VRN-H1*. The choice of *VRN-H1* allele for a specific environment should balance the vernalizing potential of the environment and the risk of frost. The winter *vrn-H1* allele can be paired with *PPD-H2* as a safeguard to promote flowering in case of incomplete vernalization, but it must also be combined with genes enhancing frost tolerance, because the rapid development linked to the functional *PPD-H2* allele could enhance frost susceptibility in facultative and winter barleys. Allele *VRN-H1-6* combines high frost tolerance and medium vernalization requirement; therefore, it could be useful for climates where the risk of frost is high but also the possibility of vernalization incompletion. To control floret survival, breeders can play with the *PPD-H1* allelic variation. The sensitive *PPD-H1* allele may gain more importance in central Europe, considering the increases in climate extremes and water and heat stresses derived from climate change. This allele is more stable in the presence of heat and drought. Regarding *VRN-H3*, the *vrn-H3c* allele showed a short LRP independently of the vernalization treatment, therefore it could be useful to escape extreme drought and heat at the end of the season. In contrast, the allele *vrn-H3d* was associated with a consistently long LRP across complete and incomplete vernalization treatments (M. Fernández-Calleja, unpublished), which could result in increased number of grains and yield under optimum conditions. Finally, *HvCEN* haplotypes offer options to modify the length of the ripening phase. A thorough understanding of the control of each stage might allow fine-tuning phenology for an optimum proportion of phases and allocation of resources, thereby, yield. Similarities and differences of the mechanisms acting in wheat and barley reveal possible new avenues for exploring further ways of fine-tuning phenology of cereal crops.

## Supplementary Information

Below is the link to the electronic supplementary material.

Supplementary file1 (XLSX 18 KB)
